# mRNA therapy corrects defective glutathione metabolism and restores ureagenesis in preclinical argininosuccinic aciduria

**DOI:** 10.1126/scitranslmed.adh1334

**Published:** 2024-01-10

**Authors:** Sonam Gurung, Oskar Vilhelmsson Timmermand, Dany Perocheau, Ana Luisa Gil-Martinez, Magdalena Minnion, Loukia Touramanidou, Sherry Fang, Martina Messina, Youssef Khalil, Justyna Spiewak, Abigail R. Barber, Richard S. Edwards, Patricia Lipari Pinto, Patrick F. Finn, Alex Cavedon, Summar Siddiqui, Lisa Rice, Paolo G.V. Martini, Deborah Ridout, Wendy Heywood, Ian Hargreaves, Simon Heales, Philippa B. Mills, Simon N. Waddington, Paul Gissen, Simon Eaton, Mina Ryten, Martin Feelisch, Andrea Frassetto, Timothy H. Witney, Julien Baruteau

**Affiliations:** 1 Great Ormond Street Institute of Child Health, University College London, London WC1N 1EH, UK; 2 School of Biomedical Engineering and Imaging Sciences, King’s College London, London SE1 7EH, UK; 3 Clinical and Experimental Sciences, Faculty of Medicine, University of Southampton, Southampton SO17 1BJ, UK; 4 Southampton NIHR Biomedical Research Centre, University Hospital Southampton NHS Foundation Trust, UK; 5 Great Ormond Street Hospital for Children NHS Foundation Trust, London, Southampton SO16 6YD, UK; 6 Santa Maria’s Hospital, Lisbon North University Hospital Center, 1649-028 Lisbon, Portugal; 7 Moderna Inc., Cambridge, MA 02139, USA; 8 Pharmacy and Biomolecular Sciences, Liverpool John Moore University, Liverpool L3 5UG, UK; 9 EGA Institute for Women’s Health, University College London, London WC1E 6HX, UK; 10 Wits/SAMRC Antiviral Gene Therapy Research Unit, Faculty of Health Sciences, University of Witswatersrand, Braamfontein 2000, Johannesburg, South Africa; 11 National Institute of Health Research Great Ormond Street Biomedical Research Centre, London WC1N 1EH, UK

## Abstract

The urea cycle enzyme argininosuccinate lyase (ASL) enables the clearance of neurotoxic ammonia and the biosynthesis of arginine. Patients with ASL deficiency present with argininosuccinic aciduria, an inherited metabolic disease with hyperammonaemia and a systemic phenotype coinciding with neurocognitive impairment and chronic liver disease. Here, we describe the dysregulation of glutathione biosynthesis and upstream cysteine utilization in ASL-deficient patients and mice using targeted metabolomics and *in vivo* positron emission tomography (PET) imaging using (*S*)-4-(3-^18^F-fluoropropyl)-L-glutamate ([^18^F]FSPG). Upregulation of cysteine metabolism contrasted with glutathione depletion and down-regulated antioxidant pathways. To assess hepatic glutathione dysregulation and liver disease, we present [^18^F]FSPG PET as a non-invasive diagnostic tool to monitor therapeutic response in argininosuccinic aciduria. Human *hASL* mRNA encapsulated in lipid nanoparticles improved glutathione metabolism and chronic liver disease. In addition, *hASL* mRNA therapy corrected and rescued the neonatal and adult Asl-deficient mouse phenotypes, respectively, enhancing ureagenesis. These findings provide mechanistic insights in liver glutathione metabolism and support clinical translation of mRNA therapy for argininosuccinic aciduria.

## Introduction

Urea cycle defects (UCDs) are inborn errors of metabolism that cause dysfunction in ammonia detoxification and endogenous arginine synthesis. Argininosuccinic aciduria (ASA) (OMIM 207900) is the second most common UCD, occurring ~1 in every 100,000 live births (1). ASA is caused by deficiency in argininosuccinate lyase (ASL), a cytosolic urea cycle enzyme, which catalyses the conversion of argininosuccinate into arginine and fumarate, thereby enabling the removal of excess nitrogen (2, 3). ASL is also involved in the citrulline-nitric oxide (NO) cycle to produce NO through the channelling of extracellular L-arginine to nitric oxide synthase (NOS) (4, 5).

Patients with ASA display acute hyperammonaemia and a chronic phenotype of neurocognitive impairment and liver disease (3). The aims of the current therapeutic guidelines for ASA are to normalise ammonia and arginine concentrations through a low-protein diet, ammonia scavenging drugs, and arginine supplementation. Liver transplantation is used in cases of progressive liver disease or recurrent hyperammonaemic crises that occur despite conventional treatment. Proposed experimental treatments include antioxidants, autophagy enhancers, creatinine supplementation, and gene therapies (2, 6–12).

Chronic liver dysfunction causes morbidity in all UCD subtypes (13, 14) but is reported with higher frequency and severity in ASA (1, 11, 13, 15). This liver disease commonly manifests with hepatomegaly and transaminitis and can progress to liver failure and, eventually, hepatocellular carcinoma (11, 14–19). Liver pathology progresses despite appropriate ammonia control, suggesting hyperammonaemia is not the sole cause (14). Other suggested mechanisms include arginine deficiency, argininosuccinate toxicity, NO deficiency, and oxidative stress (15, 16, 20). There are no reliable biomarkers that predict the degree of liver disease in ASA (16) and the underlying processes that trigger liver disease are unclear. More detailed mechanistic insight into liver pathophysiology will be crucial to identifying optimal diagnostic markers for better assessment of disease severity, prediction of disease progression, and assessment of response to therapy.

The *Asl^Neo/Neo^* mouse model recapitulates much of human ASA, with reports of hepatomegaly, elevated transaminases, aberrant hepatic glycogen accumulation, and variable fibrosis (2, 6, 9, 11, 12). Here, we studied the dysregulation of liver glutathione metabolism and its role in the chronic liver disease observed in both patients with ASA and *Asl^Neo/Neo^* mice. We assessed pathways of glutathione biosynthesis, which requires the rate-limiting biosynthetic enzyme glutamate cysteine ligase (GCL), glutathione recycling with *γ*-glutamyltranspeptidase (GGT) activity and metabolic fluxes through the hepatic antiporter system x_c_^−^, which promotes glutathione synthesis to counteract oxidative stress in health and disease (21). System x_c_^−^ activity was monitored with the positron emission tomography (PET) radiotracer (*S*)-4-(3-^18^F-fluoropropyl)-L-glutamate ([^18^F]FSPG) used both as a diagnostic tool and to assess the progression of liver disease in ASA. mRNA therapy was tested as a treatment for both neonatal and adult *Asl^Neo/Neo^* mice to restore both glutathione metabolism and ureagenesis *in vivo*.

## Results

### ASL-deficient patients and *Asl^Neo/Neo^* mice show downregulation of glutathione biosynthesis despite limited evidence of oxidative stress

**Figure 1:**
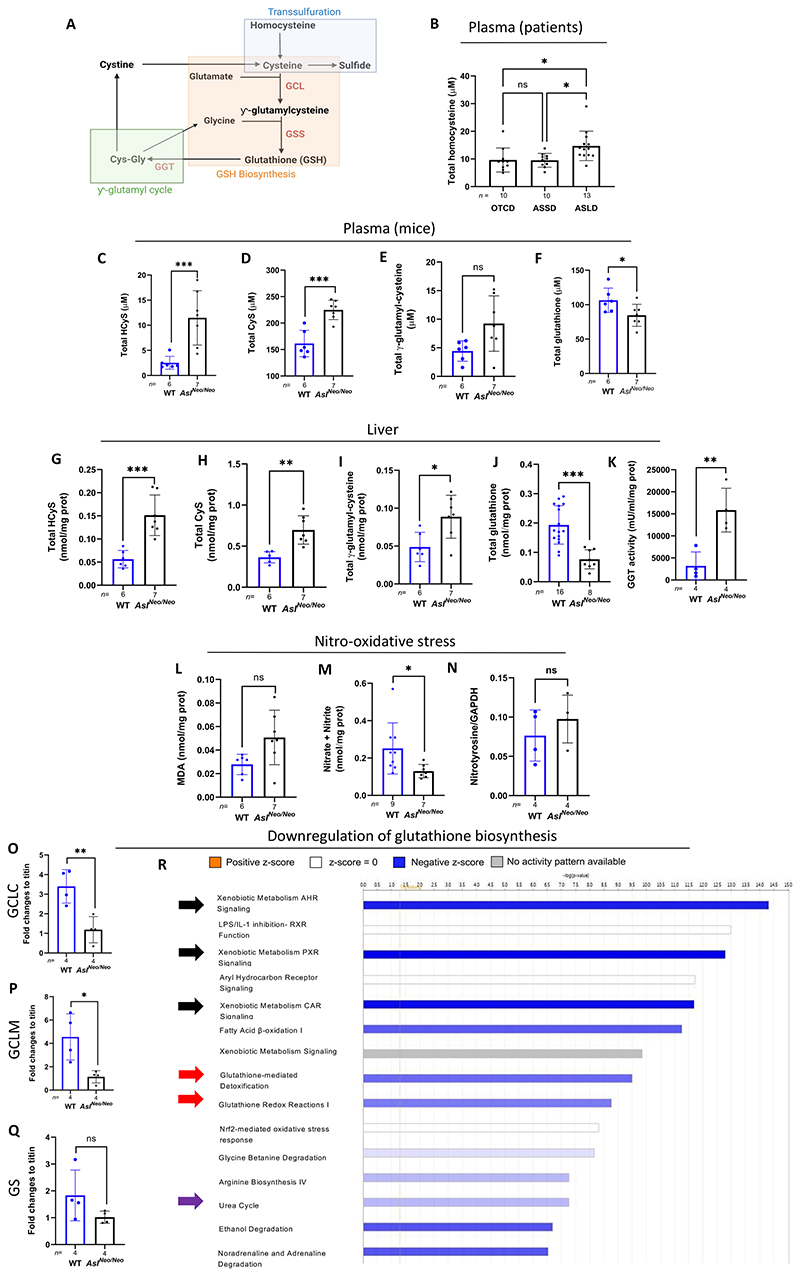
ASL-deficient patients and *Asl^Neo/Neo^* mice show dysfunction of glutathione metabolism despite limited evidence of oxidative stress. (**A**) Glutathione biosynthesis requires precursor metabolites glutamate, glycine, and cysteine, the latter being an intermediary metabolite from the transsulfuration pathway. Glutathione is degraded into cysteine-glycine by *γ*-glutamyl transferase through the *γ*-glutamyl cycle. (**B**) Mean of plasma total homocysteine in patients with OTCD (n=11-13), ASSD (n=10), ASLD (n=13). Plasma (**C**) total homocysteine, (**D**) cysteine, (**E**) *γ*-glutamyl-cysteine, (**F**) total glutathione, and liver (**G**) homocysteine, (**H**) cysteine, (**I**) *γ*-glutamyl-cysteine total thiols, and (**J**) total glutathione in 2-week old *Asl^Neo/Neo^* mice and WT littermates. (**K**) Hepatic GGT activity measured in 2-week-old *Asl^Neo/Neo^* mice and WT littermates. (**L**) Lipid peroxidation measured by thiobarbituric acid reactive substances in *Asl^Neo/Neo^* mice and WT littermates. (**M**) Nitric oxide metabolites (nitrite and nitrate) in liver samples of *Asl^Neo/Neo^* mice and WT littermates. (**N**) Quantification of liver nitrotyrosine by western blot between *Asl^Neo/Neo^* mice and WT. mRNA expression of GCL subunits (**O**) GCLC, (**P**) GCLM, and (**Q**) GS in liver of *Asl^Neo/Neo^* mice compared to WT littermates. Urea cycle dysfunction is shown by purple arrows. (**R**) Ingenuity pathway analysis of liver untargeted proteomics in *Asl^Neo/Neo^* mice compared to WT littermates, highlighting downregulation of the main glutathione functions, detoxification of xenobiotic and endogenous compounds (black arrows), antioxidant activity (red arrows). (B) One-way ANOVA with Tukey’s post-hoc test. (C) Unpaired two-tailed Student’s *t* test performed on log-transformed data. Graph displays not transformed data. (D-Q) Unpaired two-tailed Student’s *t* test; * p<0.05, ** p<0.01, *** p<0.001, ns not significant. ASSD: argininosuccinate synthase deficiency; ASLD: argininosuccinate lyase deficiency; CyS: cysteine; GCLC: glutamylcysteine ligase catalytic subunit; GCLM: glutamylcysteine ligase modifier subunit; GS: glutathione synthase; GGT: Gamma-glutamyl transferase; GSH: glutathione; HcyS: homocysteine; MDA: malondialdehyde; OTCD: ornithine transcarbamylase deficiency. Graphs show means ±SD.

Compared to other UCDs, previous publications have highlighted the role of oxidative stress in the pathophysiology of ASL deficiency (5, 9, 22). To better understand the cellular response to this oxidative stress, we explored the role of glutathione, the body’s primary antioxidant, and its regulation in ASA including its close interaction with the transsulfuration pathway (Figure 1A). We compared the plasma concentrations of total homocysteine, glycine, and glutamate in patients from Great Ormond Street Hospital for Children, London, UK who were affected by one of the 3 main urea cycle defects: ornithine transcarbamylase deficiency (OTCD; n=10), argininosuccinate synthase deficiency (ASSD; n=10), or argininosuccinate lyase deficiency (ASLD; n=13). Two liver-transplanted patients with OTCD were excluded due to normalised ureagenesis. Compared to OTCD and ASSD, patients with ASLD had significantly higher mean plasma concentrations of metabolites contributing to glutathione biosynthesis: homocysteine (Figure 1B) and glycine (Figure S1A). Plasma concentrations of glutamate, another precursor metabolite of glutathione, was not increased (Figure S1B). Plasma total homocysteine did not differ between early- and late-onset ASA (Figure S1C). Since follow-up, total plasma homocysteine (Figure S1D), glycine (Figure S1E) and glutamate (Figure S1F) concentrations remained significantly elevated for all patients with ASLD compared to those with OTCD and ASSD.

No sex difference was observed for plasma homocysteine and glycine (Table S1). Glutamate concentrations, however, were significantly elevated in females with OTCD and ASLD compared to males (Table S1). Glutamate and glycine plasma concentrations are easily influenced by diet, medications, metabolic control, and delayed analytical processing. Clinical information regarding these patients with OTCD, ASSD, and ASLD are presented in Table S2. Samples collected during hyperammonemic episodes were excluded from data collection. Patients with average total homocysteine above the normal upper range limit were not screened for methyltetrahydrofolate (*MTHFR*) polymorphism, which may contribute to the differences observed here between patient types. No vitamin B12 deficiency was observed in the patients (Figure S1G).

We measured the metabolites of the glutathione pathway in the hypomorphic *Asl^Neo/Neo^* mouse model, which recapitulates much of the human phenotype of ASLD (4, 9). As sulfur-containing amino acids (and H_2_S) exist in different forms (23), we measured both total and reduced free thiol form (R-SH) to determine any compromise to the cellular antioxidant buffering capacity. Corroborating human data, plasma total (Figure 1C) and free (Figure S2A) homocysteine concentrations in 2-week-old *Asl^Neo/Neo^* mice were significantly elevated compared to WT littermates. Other plasma metabolites of the glutathione biosynthesis pathway, total (Figure 1D) and free (Figure S2B) cysteine were also significantly increased in *Asl^Neo/Neo^* mice compared to WT. Total *γ*-glutamyl-cysteine (Figure 1E) and free *γ*-glutamyl-cysteine (Figure S2C) showed no difference. In contrast, plasma total glutathione was significantly decreased (Figure 1F). Most of the glutathione released in the bloodstream is synthesised in the liver (24). Similar to plasma, liver concentrations of total (Figure 1G) and free (Figure S2D) homocysteine, total (Figure 1H) and free (Figure S2E) cysteine, and total *γ*-glutamyl-cysteine (Figure 1I) showed a significant increase in *Asl^Neo/Neo^* mice compared to WT. Liver free *γ*-glutamyl-cysteine (Figure S2F) showed no difference. As in plasma, total glutathione concentrations in liver were significantly decreased but to a far greater extent (Figure 1J).

Total glutathione is degraded by GGT at the external surface of epithelial cells (25). GGT activity and expression in liver showed a significant 5-fold increase in *Asl^Neo/Neo^* mice compared to WT (Figure 1K and Figure S2G). Total cysteine-glycine synthesised from glutathione catabolism by GGT was also increased in liver (Figure S2H), although free cysteine-glycine was unchanged (Figure S2I). *Asl^Neo/Neo^* mice (4) and patients with ASA (26, 27) may present chronic kidney disease and renal failure. Supporting this, urine glutathione concentrations were significantly increased in *Asl^Neo/Neo^* mice compared to WT, suggesting defective renal glutathione reabsorption (Figure S2J). Urine cysteine concentrations were also significantly increased in *Asl^Neo/Neo^* mice compared to WT (Figures S2K), in line with an elevation of these metabolites in the plasma and livers, a consequence which is partially explained by increased GGT activity. Urine cysteine-glycine concentrations, however, were not significantly increased (Figure S2L).

The increase of free thiols metabolites suggested that the persisting buffering capacity and protective role against oxidative stress of free thiols (23, 28) was maintained both systemically and in the liver. To better characterise the oxidative stress in ASA, we assessed the ratio of reduced (GSH) versus oxidised glutathione (GSSG), whereby a reduction in this ratio is a common marker of oxidative stress (29). Here the liver GSH:GSSG ratio did not differ between *Asl^Neo/Neo^* and WT littermates (Figure S2M), indicating that the reduction of total glutathione pool in *Asl^Neo/Neo^* mice was not associated with increased glutathione oxidation. The comparison of the different steady-state concentrations of the precursors and breakdown products of glutathione suggested that precursors accumulated due to a bottleneck in one of the rate-limiting steps of glutathione biosynthesis, whereas glutathione catabolism by GGT was enhanced, explaining the lower glutathione concentrations in both the circulation and in the liver of *Asl^Neo/Neo^* mice.

To determine whether oxidative stress might further contribute to the lower concentrations of total glutathione in plasma and liver of *Asl^Neo/Neo^* mice, we measured lipid peroxidation products in the liver using the thiobarbituric acid reactive substances (TBARS) assay. Lipid peroxidation was not significantly increased in *Asl^Neo/Neo^* mice versus WT (Figure 1L). The steady-state concentration of the oxidative breakdown products of NO, nitrite (NO_2_^-^) and nitrate (NO_3_^-^), were significantly lower in plasma (Figure S2N) and liver (Figure 1M), as previously reported in this disorder (4, 5). Nitroso-species showed no difference between *Asl^Neo/Neo^* livers versus WT, and did not support NO deficiency as a critical pathophysiological mechanism in the liver (Figure S2O). The lack of difference in nitrotyrosine concentrations by western blot in *Asl^Neo/Neo^* livers versus WT confirmed the absence of nitro-oxidative stress (Figure 1N, Figure S2P). The nuclear factor erythroid 2-related factor 2 (Nrf2) is a transcriptional factor regulating antioxidant response against oxidative stress. Nrf2 protein abundances did not differ between WT and *Asl^Neo/Neo^* livers (Figure S2Q). Taken together, upstream precursors of glutathione biosynthesis accumulated in ASA, coinciding with reduced total glutathione, compromised NO production, moderate oxidative stress, and persisting antioxidant capacity.

Glutathione biosynthesis relies on 2 enzymatic steps: the rate limiting glutamate cysteine ligase (GCL) catalyses the conversion of glutamate and cysteine to *γ*-glutamyl-cysteine, and then glutathione synthase (GS) catalyses the conversion of *γ*-glutamyl-cysteine and glycine to glutathione. GCL is a heterodimer with a heavy catalytic subunit (GCLC) and a light or modifier subunit (GCLM) (25). We hypothesised that reduced glutathione was not a consequence of increased oxidative stress, but an effect of deficiencies in the enzymes involved in its biosynthesis. Indeed, *Asl^Neo/Neo^* mice had decreased gene expression of GCLC (Figure 1O) and GCLM (Figure 1P) compared to WT, whereas GS expression showed no significant difference (Figure 1Q). Supporting this, untargeted proteomics comparing WT and *Asl^Neo/Neo^* livers highlighted glutathione function as downregulated (Figure 1R, Figure S3A-C).

### A non-invasive marker confirms impaired glutathione metabolism in *Asl^Neo/Neo^* mice

**Figure 2:**
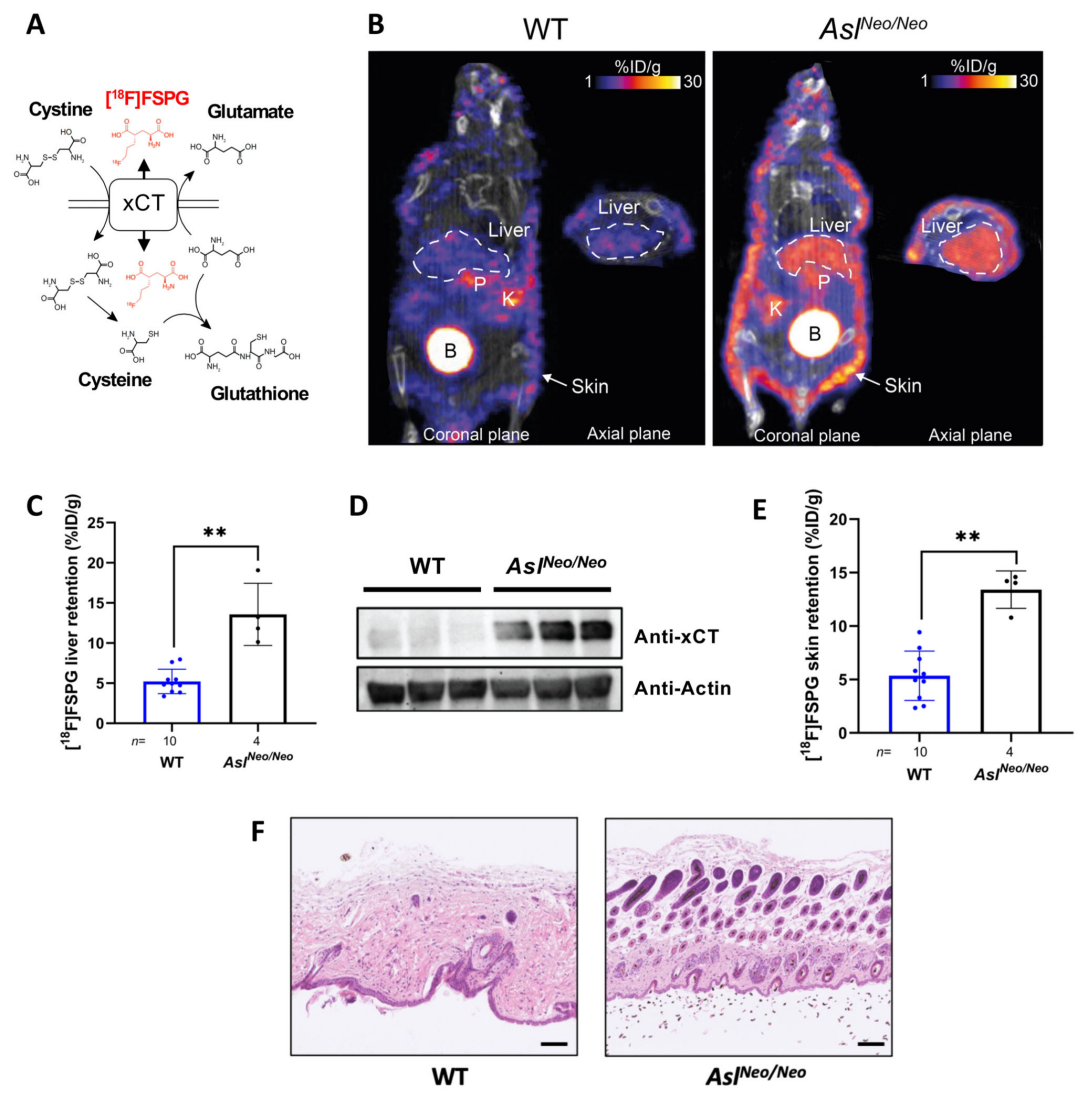
A non-invasive marker confirms the impaired glutathione metabolism in *Asl^Neo/Neo^* mice. **(A)** Schematic overview of system x_c_^−^ function, shuttling cystine, glutamate and [^18^F]FSPG (red) across the cell membrane. Reduced cystine, cysteine, and glutamate are precursors for glutathione biosynthesis. **(B)** Representative PET/CT images of [^18^F]FSPG distribution (%ID/g) in the coronal and axial plane of 2 week old WT littermates and *Asl^Neo/Neo^* mice. P=pancreas, B=bladder, K=kidney. **(C)** Quantified [^18^F]FSPG retention in the liver 60 min post-injection. **(D)** Western blot of liver xCT expression of WT and *Asl^Neo/Neo^* mice. **(E)** Quantified [^18^F]FSPG retention in the skin of WT and *Asl^Neo/Neo^* mice 60 min post-injection. (**F**) H&E stain from skin of Asl^Neo/Neo^ and WT mice showing architectural differences highlighting the skin abnormality observed in ASL deficiency. Scale bar is 100 *μ*m. (C, E) Unpaired 2-tailed Student’s *t* test; **p<0.01. Graphs show mean ±SD.

The biosynthesis of glutathione is dependent on cellular import of cystine in exchange for glutamate efflux via the cystine/glutamate antiporter system x_c_^−^, a transmembrane transport system (Figure 2A) (31). Cystine is subsequently reduced to cysteine for *de novo* glutathione biosynthesis (25). Metabolic reprogramming in cancer cells generate oxidative stress, which is balanced by increased glutathione biosynthesis via enhanced x_c_^−^ mediated cystine import (30). Using positron emission tomography (PET), the radiolabelled glutamate analogue [^18^F]FSPG provides an *in vivo* functional readout of *de novo* glutathione biosynthesis and has been used both in the clinic for cancer diagnosis (31–33), and preclinically to assess cancer drug resistance (34) and disease progression in multiple sclerosis (35).

To functionally assess alterations in the glutathione biosynthetic pathway, [^18^F]FSPG was administered intravenously (IV) to 2-3 weeks-old *Asl^Neo/Neo^* mice and WT littermates, with radiotracer retention dynamically imaged by PET. In all mice, typical healthy tissue [^18^F]FSPG retention was observed in the salivary glands, thymus, and pancreas, accompanied by renal elimination (Figure 2B). Liver [^18^F]FSPG retention for *Asl^Neo/Neo^* mice was 14 ± 4% injected dose (ID)/g, which was 3-fold higher (*p*=0.002) than that of WT mice (5.2 ± 1.5% ID/g). In PET images of WT mice, [^18^F]FSPG retention in the liver was just above background, with images dominated by radiotracer retention in the pancreas and kidney. Conversely, it was challenging to distinguish between pancreatic and liver [^18^F]FSPG retention in *Asl^Neo/Neo^* mice (Figures 2B, 2C, Figure S4A). We confirmed that the protein expression of xCT in 2-week-old *Asl^Neo/Neo^* mouse liver was substantially increased compared to WT littermates, which had low baseline expression (Figure 2D). High [^18^F]FSPG retention was also present in the skin of *Asl^Neo/Neo^* mice (13 ± 1.8% ID/g) which was not the case in WT littermates (5.3 ± 2.3% ID/g; Figure 2B, 2E; *p*=0.002; Figure S4B). Increased [^18^F]FSPG skin retention accompanied gross changes to tissue structure, as shown by hematoxylin and eosin (H&E) staining (Figure 2F). [^18^F]FSPG is therefore a useful non-invasive marker of aberrant glutathione metabolism in the liver and skin of *Asl^Neo/Neo^* mice, mediated at least in part through the upregulated expression of the xCT antiporter.

### Single intravenous administration of *hASL* mRNA corrects ureagenesis up to 7 days in adult *Asi^Neo/Neo^* mice

**Figure 3:**
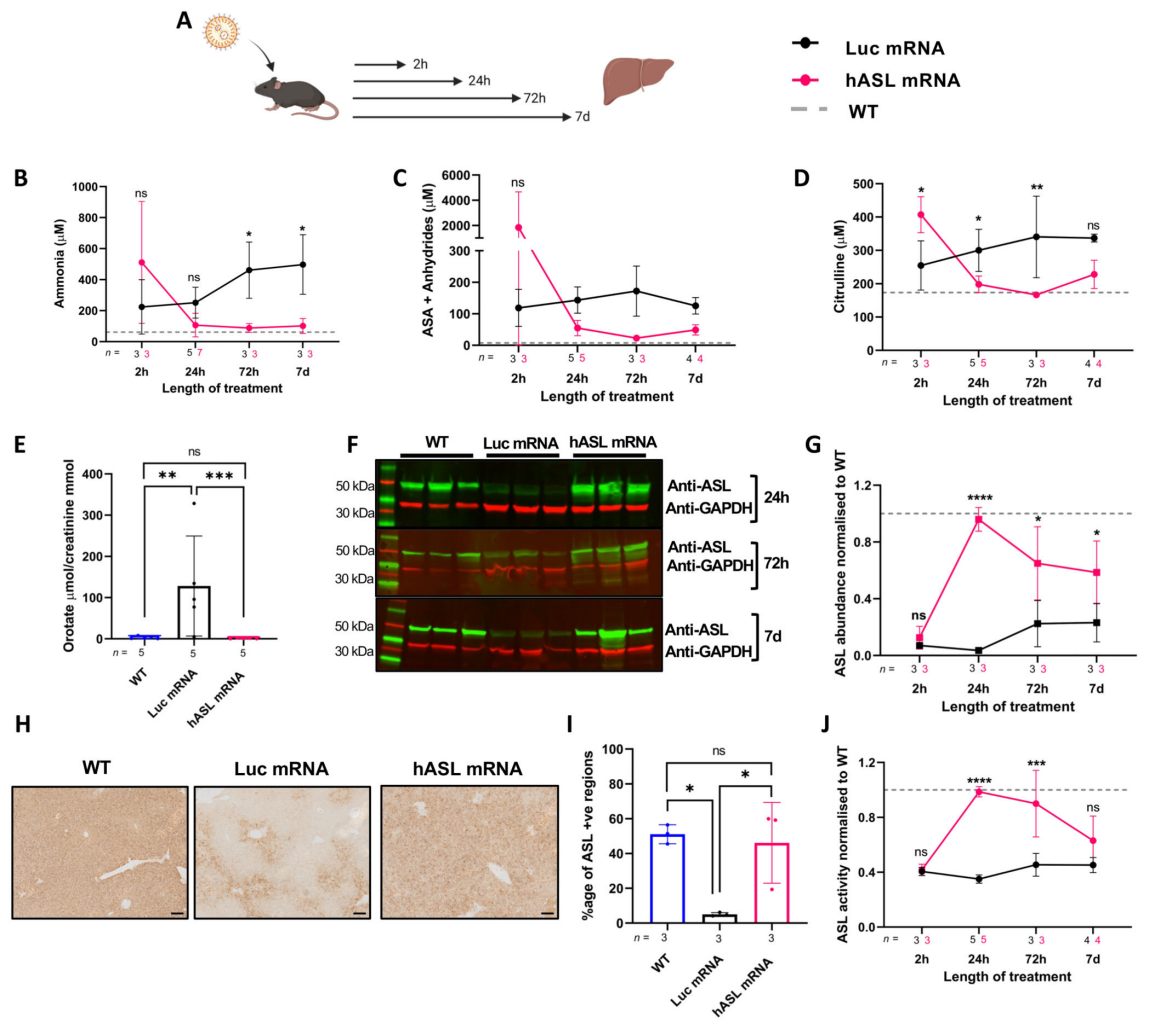
Single intravenous administration of *hASL* mRNA corrects ureagenesis up to 7 days in adult *Asl^Neo/Neo^* mice **(A**) Schematic illustration of experimental plan. Three-week old *Asl^Neo/Neo^* mice received a single intravenous (IV) injection of either *hASL* or *Luc* mRNA at 1mg/kg body weight and were sacrificed at 2h, 24h, 72h, or 7 days. **(B**) Average ammonia concentrations from plasma and average (**C**) argininosuccinic acid (**D**) citrulline concentrations from dried blood spots at 2, 24, 72 hours, or 7 days. (**E**) Urine orotic acid concentrations normalised to creatinine at 24 hours. (**F**) ASL liver western blot at 24 hours, 72 hours, and 7 days. (**G**) Quantification of ASL immunoblot normalised to GAPDH (**H**) Representative images of liver ASL immunostaining at 24 hours post-mRNA administration and (**I**) Quantification. Scale bar= 100μM. (**J**) Liver ASL activity at 2, 24, 72 hours, and 7 days. Values normalised against WT control. (B, D, G, J) Two-way ANOVA with Šídák’s post-hoc test per timepoint. Grey dotted line represents mean WT values. (C) Two-way ANOVA with Šídák’s post-hoc test per timepoint post log transformation, (E) One-way ANOVA post Tukey’s post-hoc test comparison post log-transformation (I) One-way ANOVA post Tukey’s post-hoc test comparison, ns=not significant, *p<0.05, **p<0.01, ***p<0.005, ****p<0.0001. Graphs show mean ±SD.

The promising therapeutic effects of mRNA technology have been demonstrated recently in multiple liver inherited metabolic conditions (36, 37). *hASL* mRNA encapsulated in lipid nanoparticles were specifically engineered with *hASL* codon and amino acid sequence optimisation. *hASL* mRNA restored ASL expression (fig. S5A, B) and activity (fig. S5C) in ASL-deficient fibroblasts from patients with ASA compared to a *Luciferase (Luc)* mRNA control lipid nanoparticle. mRNA therapy has transient efficacy and requires re-administration to enable a sustained effect. Thus, we conducted a pharmacokinetic study of *hASL* mRNA in *Asl^Neo/Neo^* mice to assess the efficacy and duration of effect on the urea cycle. Three-week-old *Asl^Neo/Neo^* mice received a single IV injection of either *hASL* or *Luc* mRNA at 1 mg mRNA/kg body weight and were sacrificed at 2h, 24h, 72h, or 7 days (Figure 3A). Due to the severity of the phenotype, the experimental design did not include any longitudinal assessment of biomarkers to avoid additional stress that could precipitate the animal’s death. Specific cohorts of animals received mRNA therapy at T0 and were harvested at the selected time point. A marked reduction of plasma ammonia (Figure 3B), argininosuccinic acid (Figure 3C), and citrulline (Figure 3D) in dried blood spots as well as urinary orotate (Figure 3E) were observed within 24h of administration in *hASL* mRNA-treated *Asl^Neo/Neo^* mice compared to the control (*Luc* mRNA)-treated group. This effect was sustained over seven days. The concentrations of these metabolites post-*hASL* mRNA treatment in *Asl^Neo/Neo^* mice were comparable to the physiological concentrations in WT mice. Plasma ammonia, argininosuccinic acid, and citrulline in dried blood spots were elevated at 2 hours post-injection in the *hASL* mRNA versus *Luc* mRNA treated group (Figure 3A-C), raising the question of a risk of acute impairment of the urea cycle. No effect on arginine concentrations was observed (Figure S6A). Western blot and immunohistochemistry data in liver showed restored ASL protein abundance at physiological levels at 24h post-administration of *hASL* mRNA (Figures 3F-I, Figure S6B-D). ASL abundance was consistently higher in *hASL-*treated versus the *Luc* mRNA-treated group over this seven-day time course. Liver ASL activity was also restored to physiological levels at 24h and 72h in the *hASL*-treated compared to the *Luc* mRNA-treated group but began to decline by seven days (Figure 3J).

### *hASL* mRNA therapy from birth normalises the phenotype of *Asl^Neo/Neo^* mice

**Figure 4:**
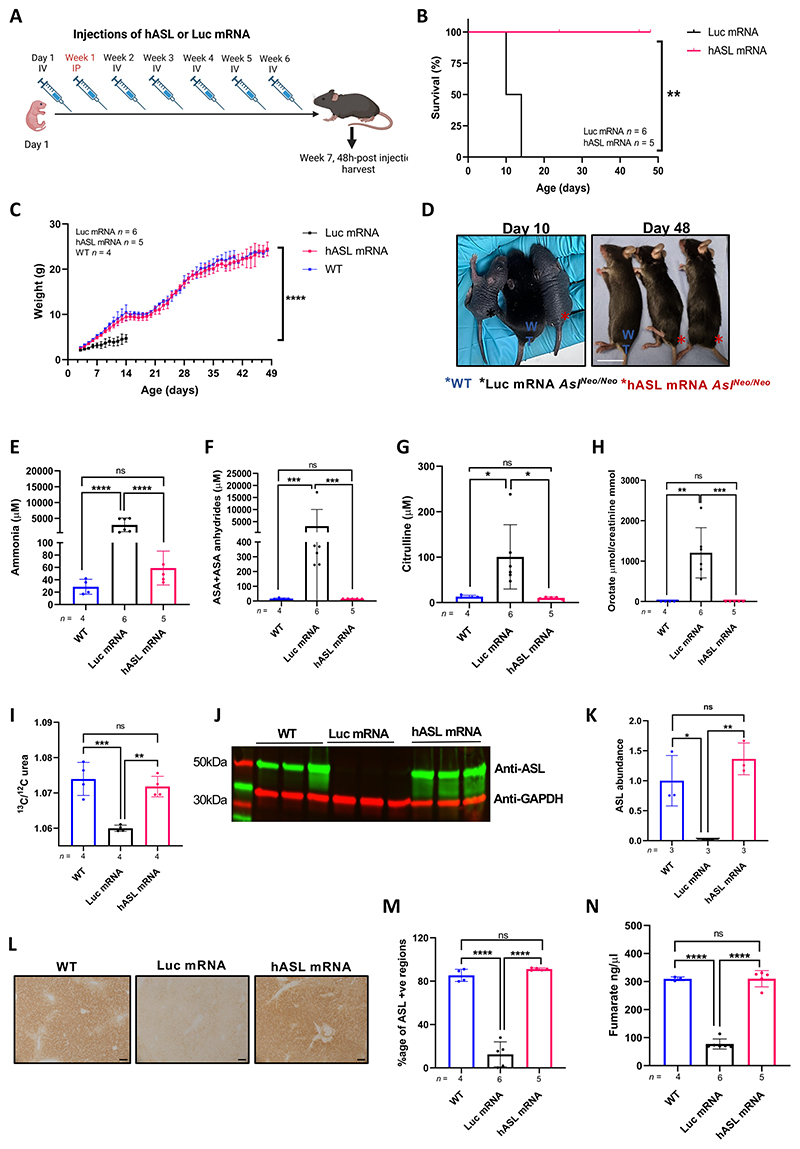
*hASL* mRNA therapy from birth normalises the phenotype of *Asl^Neo/Neo^* mice. (**A**) Schematic illustration of experiment. *Asl^Neo/Neo^* mice were given weekly intravenous (IV) dose of 1mg/kg of either *hASL* or *Luc* mRNA from birth up to 7 weeks, except for week 1 where the mice were administered intraperitoneally with dose of 2 mg/kg. Harvest was performed 48 hours after the last injection. (**B**) Kaplan-Meier survival curve of *hASL*, and *Luc* mRNA-treated *Asl^Neo/Neo^* mice. (**C**) Average growth curve of WT, *hASL*, and *Luc* mRNA-treated *Asl^Neo/Neo^* mice. (**D**) Representative images of wild-type (blue asterisk), *hASL* (red asterisk), and *Luc* mRNA-treated (black asterisk) *Asl^Neo/Neo^* mice at harvest. Scale bar=2cm. (**E**) Average plasma ammonia, (**F**) argininosuccinic acid (**G**), and citrulline concentrations from dried blood spots, (**H**) urinary orotic acid, and (**I**) C13 ureagenesis from WT, *hASL*, and *Luc* mRNA-treated *Asl^Neo/Neo^* mice. (**J**) ASL western blot of WT, *hASL*, and *Luc* mRNA-treated *Asl^Neo/Neo^* mice and (**K**) quantification. (**L**) Representative images of ASL immunostaining in livers of WT, *hASL*, and *Luc* mRNA-treated *Asl^Neo/Neo^* mice (scale bar=100μM) and (**M**) quantification. (**N**) Liver ASL activity from WT, *hASL*, and *Luc* mRNA-treated *Asl^Neo/Neo^* mice livers. (B) Log-rank (Mantel-Cox). (C) Simple linear regression analysis comparing average slopes per group. (E, G-I, K, M, N) One-way ANOVA with Tukey’s post-hoc test analysis, (F) One-way ANOVA post Tukey’s post-hoc test comparison on log-transformed values, ns=not significant, *p<0.05, **p<0.01, ***p<0.005, ****p<0.0001. Graphs show mean ±SD.

Pharmacodynamic data showed a single mRNA dose to be efficacious for up to seven days. To better understand the value of this treatment, we initiated a survival study with repeated administration of *hASL* mRNA versus *Luc* mRNA in neonatal *Asl^Neo/Neo^* pups. Mice received systemic administration of mRNA constructs every seven days with the first IV dose administered at day 1 of life. Due to difficulties injecting young pups, an intraperitoneal (IP) dose of 2 mg/kg at day 8 of life was performed. This twice higher dose was based on liver biodistribution between IV and IP routes, which showed that a two-fold higher IP dose has a similar liver biodistribution as a single IV dose (Figures S7A, 7B). Mice were treated for seven weeks and harvested 48 h after the last injection (Figure 4A). The macroscopic phenotype of *Asl^Neo/Neo^* mice was restored to that of WT littermates in the *hASL* mRNA treatment group, with normalisation of survival (Figure 4B, *p*=0.002), growth (Figure 4C, Figure S8A), fur (Figure 4D), and hepatomegaly (Figure S8B). One *hASL* mRNA treated mutant was culled at 24 days of age due to malocclusion, a complication unrelated to the ASL phenotype or mRNA therapy, and was excluded from the analysis. In contrast, *Luc* mRNA-treated *Asl^Neo/Neo^* littermates showed abnormal fur, impaired growth, and early death within 2 weeks of life (Figure 4B-D).

Animals which survived seven weeks were culled 48 h after the last mRNA injection. Analysis showed normalization of ammonaemia (Figure 4E), argininosuccinic acid (Figure 4F), and citrulline (Figure 4G) concentrations in dried blood spots, and urinary orotate concentrations (Figure 4H) in *hASL* mRNA-treated *Asl^Neo/Neo^* mice. No significant differences in arginine concentrations in dried blood spots between WT or *Luc* mRNA- or *hASL* mRNA-treated *Asl^Neo/Neo^* mice were observed (Figure S8C). Elevated plasma amino transferase (ALT) was normalized in the *hASL* mRNA-treated *Asl^Neo/Neo^* mice (Figure S8D). Longitudinal analysis of plasma ammonia (Figure S8E), argininosuccinic acid, (Figure S8F), and citrulline (Figure S8G) concentrations in dried blood spots, and urinary orotate concentrations (Figure S8H) in *hASL* mRNA-treated *Asl^Neo/Neo^* mice showed sustained therapeutic benefit over time. Next, functional assessment of urea cycle *in vivo* was measured by quantifying labelled urea in the plasma following the systemic injection of the ^13^C labelled sodium acetate stable isotope 30 minutes pre-harvest. ^13^C labelling showed restored ureagenesis in the *hASL* treated *Asl^Neo/Neo^* mice (Figure 4I). ASL levels in liver assessed by western blot (Figures 4J, 4K) and immunohistochemistry (Figures 4L, 4M) were restored to physiological levels and patterns after *hASL* mRNA therapy. Similar to single-dose short-term therapy, ASL activity in liver was restored to WT physiological levels after *hASL* mRNA therapy in *Asl^Neo/Neo^* mice at this late time-point (Figure 4N). Gender analysis showed that there were no sex-related differences in the different efficacy endpoints in the *hASL* mRNA-treated group (Table S3).

### *hASL* mRNA therapy partially rescues the adult phenotype in *Asl^Neo/Neo^* mice

**Figure 5:**
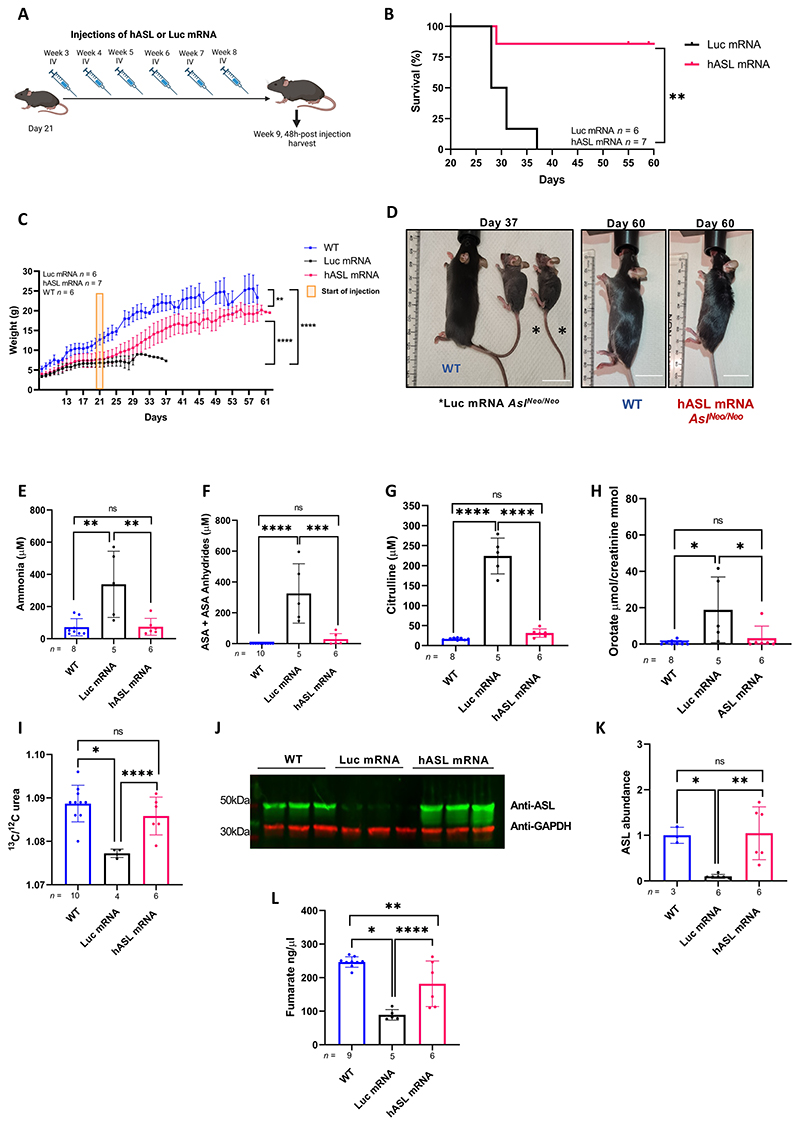
*hASL* mRNA therapy partially rescues the adult phenotype in *Asl^Neo/Neo^* mice. (**A**) Schematic illustration of experimental plan. *Asl^Neo/Neo^* mice were given weekly intravenous (IV) dose of 1mg/kg of either *hASL* or *Luc* mRNA from day 21 up to 9 weeks. (**B**) Kaplan-Meier survival curve of *hASL* and *Luc* mRNA-treated *Asl^Neo/Neo^* mice. (**C**) Average growth curve of WT, *hASL*, and *Luc* mRNA-treated *Asl^Neo/Neo^* mice. (**D**) Representative images of WT, *hASL*, and *Luc* mRNA-treated *Asl^Neo/Neo^* mice at harvest. Scale bar=2cm. (**E**) Average plasma ammonia concentration, (**F**) argininosuccinic acid, (**G**) and citrulline concentrations from dried blood spots, (**H**) urinary orotic acid, and (**I**) C13 ureagenesis from WT, *hASL*, and *Luc* mRNA-treated *Asl^Neo/Neo^* mice. (**J**) ASL western blot of WT, *hASL*, and *Luc* mRNA- treated *Asl^Neo/Neo^* mice and (**K**) quantification. (**L**) Liver ASL activity from WT, *hASL*, and *Luc* mRNA-treated *Asl^Neo/Neo^* mice livers. (B) Log-rank (Mantel-Cox), *p*=0.0025. (C) Simple linear regression analysis comparing average slopes per group. (F-I, K, L) One-way ANOVA with Tukey’s post-hoc test analysis. (E) One-way ANOVA with Tukey’s post-hoc test analysis on log-transformed values ns=not significant, *p<0.05, **p<0.01, ****p<0.0001. Graphs show mean ±SD.

As untreated *Asl^Neo/Neo^* mice start dying from 2-3 weeks of age (4), we next wanted to assess whether it was possible to rescue of the severe phenotype of *Asl^Neo/Neo^* mice after late initiation of *hASL* mRNA therapy. *Asl^Neo/Neo^* mice received their first IV *hASL* mRNA dose in early adulthood at day 21 of life followed by weekly mRNA administration for up to 8 weeks (Figure 5A). All but one treated mouse survived to the end of the study which died after two injections at day 31 of life. In contrast, *Luc* mRNA-treated mice only survived up to day 37 of life, with most animals dying before day 30 (Figure 5B, *p*=0.002). *hASL* mRNA-treated *Asl^Neo/Neo^* mice showed significantly improved growth compared to *Luc* mRNA *Asl^Neo/Neo^* littermates, however the body weight remained significantly lower than WT littermates (Figure 5C, Figure S9A). The full recovery of hair growth in *hASL* mRNA-treated *Asl^Neo/Neo^* mice had a similar fur pattern compared to WT (Figure 5D). Liver to body weight ratio remained significantly elevated in both treated *Asl^Neo/Neo^* mice groups (Figure S9B). Plasma ammonia (Figure 5E), argininosuccinic acid (Figure 5F), and citrulline (Figure 5G) in dried blood spots and urinary orotic acid concentrations (Figure 5H) were reduced after *hASL* mRNA therapy to physiological WT concentrations. In pups treated soon after birth, ^13^C ureagenesis revealed restored *in vivo* urea cycle function comparable to that of WT in *hASL* mRNA-treated *Asl^Neo/Neo^* mice (Figure 5I) compared to *Luc* mRNA-treated *Asl^Neo/Neo^* littermates. There were no significant differences in arginine concentrations between WT, *Luc* mRNA and *hASL* mRNA-treated *Asl^Neo/Neo^* groups (Figure S9C). ALT concentrations indicated an absence of liver toxicity in the *hASL* mRNA-treated *Asl^Neo/Neo^* mice compared to WT littermates (Figure S9D). ASL concentrations in the liver, assessed by western blot (Figures 5J, 5K), were restored to physiological concentrations after *hASL* mRNA therapy compared to WT. Liver ASL activity was also significantly improved following *hASL* mRNA compared to *Luc* mRNA in *Asl^Neo/Neo^* mice (Figure 5L). Gender analysis could not determine any sex differences between the different efficacy endpoints in the *hASL* mRNA-treated group (Figure S10 and Table S4).

### [^18^F]FSPG-PET provides a sensitive pharmacodynamic marker of *hASL* mRNA treatment

**Figure 6:**
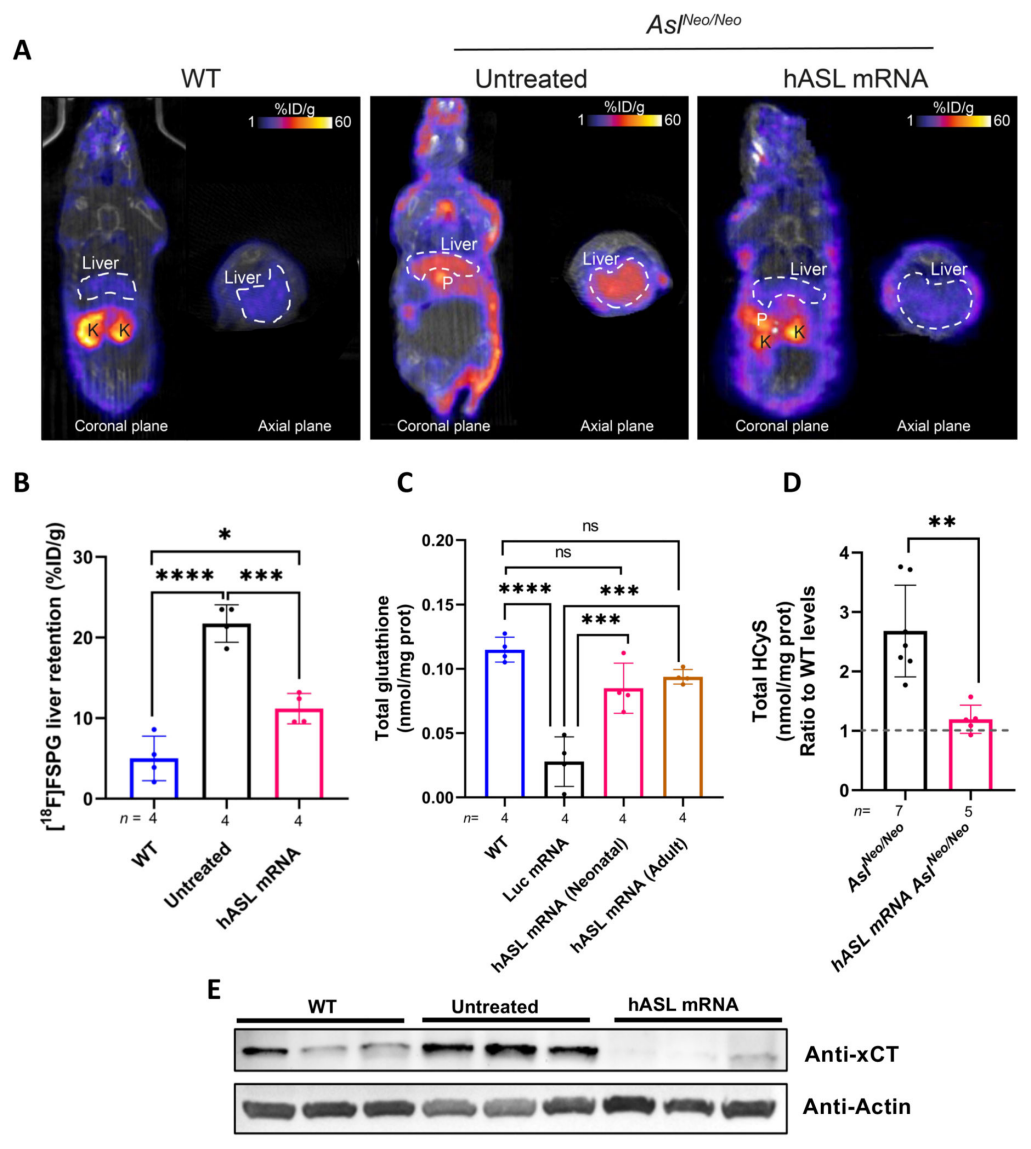
*hASL* mRNA therapy corrects the dysfunction of glutathione metabolism in *Asl^Neo/Neo^* mice. **(A)** [^18^F]FSPG distribution (%ID/g) in representative coronal and axial plane PET/CT images of WT, untreated *Asl^Neo/Neo^*, and *hASL* mRNA-treated *Asl^Neo/Neo^* mice. K= Kidney, P= Pancreas. **(B)** [^18^F]FSPG quantification of the liver in WT, untreated *Asl^Neo/Neo^*, and *hASL* mRNA-treated *Asl^Neo/Neo^* mice 60 min post-injection. **(C**) Western blot of xCT expression in untreated *Asl^Neo/Neo^* liver and liver of *hASL* mRNA-treated *Asl^Neo/Neo^* mice. **(D)** Total glutathione concentrations from liver in WT, Luc mRNA-treated *Asl^Neo/Neo^* mice, and *hASL* mRNA-treated *Asl^Neo/Neo^* mice from neonatal or adulthood. **(E)** Liver total homocysteine concentrations expressed as ratio relative to WT (shown as dotted line) from untreated versus *hASL* mRNA-treated *Asl^Neo/Neo^* adult mice. Graph shows mean ±SD. (B, D) One-way ANOVA with Tukey’s post-hoc test; (E): Unpaired 2-tailed Student’s *t* test; ns=not significant, **p<0.01, ***p<0.001, ****p<0.0001.

To investigate the potential of [^18^F]FSPG PET as a non-invasive tool of therapeutic efficacy, [^18^F]FSPG was administered IV to 2 weeks-old untreated and *hASL* mRNA-treated *Asl^Neo/Neo^* mice (IV administration of 1 mg/kg *hASL* mRNA at birth followed by weekly IP administration of 2 mg/kg mRNA before imaging at 2 weeks of age). Supporting our functional and metabolic data, [^18^F]FSPG retention was halved in *hASL* mRNA-treated (11 ± 2.0% ID/g) versus untreated *Asl^Neo/Neo^* mice (22 ± 2.3% ID/g; *p* = 0.026; Figure 6A, 6B, Figure S11A). At the treatment time-point, however, [^18^F]FSPG retention was not completely restored to baseline levels seen in WT livers (5.0 ± 2.8% ID/g). In line with lowered [^18^F]FSPG retention, glutathione metabolism was corrected, with restoration of hepatic liver glutathione in both neonatal and adult treated *Asl^Neo/Neo^* mice to concentrations similar to those of WT mice (Figure 6C). This restoration of glutathione was associated with a significant reduction of total homocysteine ratio compared to WT in livers from *hASL* mRNA-treated versus untreated *Asl^Neo/Neo^* mice (Figure 6D). In these animals, the expression of cystine/glutamate antiporter system x_c_^−^ was greatly reduced in livers from *hASL* mRNA-treated versus *Asl^Neo/Neo^* mice (Figure 6E). Conversely, skin [^18^F]FSPG retention was not affected by mRNA therapy. [^18^F]FSPG skin retention was 4.2 ± 3.4% ID/g in WT mice, compared to 15 ± 3.7% ID/g and 15 ± 4.2% ID/g in untreated and *hASL* mRNA treated *Asl^Neo/Neo^* mice, respectively (Figures S11B, S11C).

### *hASL* mRNA therapy corrects the metabolic dysfunction and liver pathophysiology in *Asl^Neo/Neo^* mice

**Figure 7:**
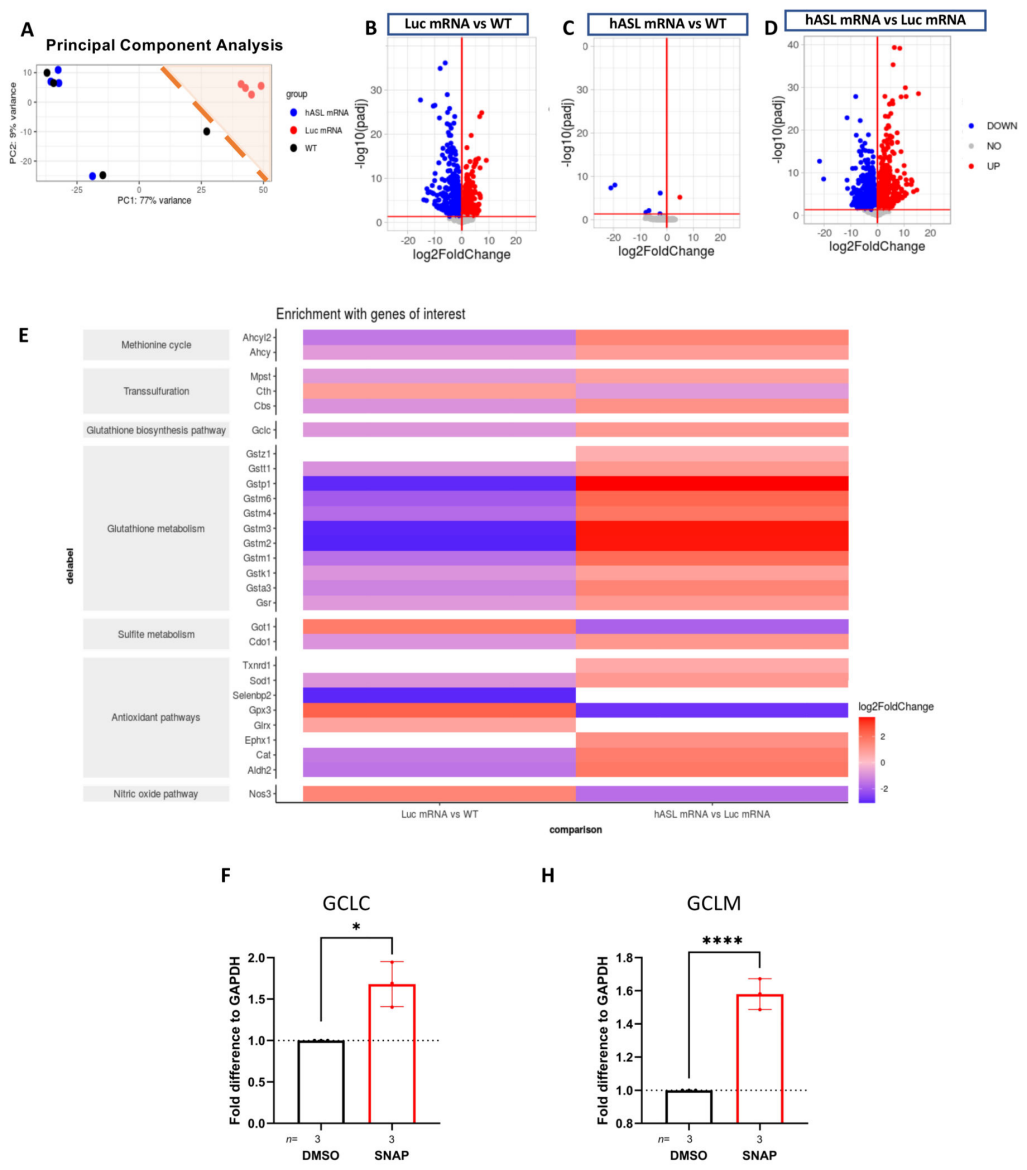
*hASL* mRNA therapy corrects metabolic dysfunction and liver pathophysiology in *Asl^Neo/Neo^* mice. (**A**) Principal component analysis plots comparing treatment applied (untreated WT, *hASL* mRNA, or *Luc* mRNA) and mouse genotype (WT or *Asl^Neo/Neo^)* with percentage of variance associated with each axis. (**B**) Volcano plots showing differential gene expression (DEG) analysis of *Luc* mRNA vs WT, (**C**) *hASL* mRNA vs WT, and (**D**) *hASL* mRNA vs *Luc* mRNA. Scatter plots show log-transformed adjusted p-values (<0.05) on the y-axis against log2 fold change (>0.10) values on the x-axis. Blue and red dots represent genes that were significantly (FDR-corrected p-value of <0.05) downregulated and upregulated, respectively, between groups. Grey dots represent genes that were not significantly altered. (**E**) Pathway analysis highlighting genes of interest significantly altered in DEG analysis organised with their associated pathways when comparing *Luc* mRNA vs WT and *hASL* mRNA vs *Luc* mRNA groups. mRNA expression of **(F)** *GCLC* and **(G)** *GCLM* with NO donor SNAP at 200μM versus control DMSO in Huh7 cells. (**F, G**): Unpaired 2-tailed Student’s *t* test; ns=not significant, ***p<0.001, ****p<0.0001; average values from 3 independent experiments. Each dot represents one experiment.

We wanted to determine the extent of the correction of liver metabolic dysfunction after *hASL* mRNA treatment in *Asl^Neo/Neo^* mice. To do this, we used RNA-sequencing (RNA-seq) transcriptomic analysis and visualised the overall variation in gene expression across WT and *Asl^Neo/Neo^* mice treated at birth with either *hASL* or *Luc* mRNA therapy. Principal component analysis showed clustering of the *hASL* mRNA-treated and WT liver samples, suggesting a similar profile of gene expression in both groups. In contrast, *Luc* mRNA treated *Asl^Neo/Neo^* mice clustered separately (Figure 7A). Next, we analysed differential gene expression identifying all genes with a log2-fold change of >0.1 or < -0.1 and passing an FDR cut off of <0.05. Comparing WT vs *Luc* mRNA *Asl^Neo/Neo^* groups, we found 2,705 genes to be significantly up- (1,257 genes) or down-regulated (1,448 genes; Figure 7B). Only 7 genes (1 up-regulated and 6 down-regulated) were differentially expressed between WT vs *hASL* mRNA *Asl^Neo/Neo^* mice livers, thereby demonstrating the efficacy of mRNA therapy in correcting liver dysfunction (Figure 7C). No pathway correlation was observed between these genes, which had different localisation and function (Table S5). This interpretation of the data was supported by the analysis of differential gene expression between mRNA and *hASL* mRNA *Asl^Neo/Neo^*, which similarly to the WT vs *Luc* mRNA *Asl^Neo/Neo^* comparison, we identified a large number of differentially expressed genes (4,297 genes): 1,962 genes being significantly up-regulated (log2-fold change > 0.1 and FDR < 0.05) and 2,335 being significantly down-regulated (log2-fold change < -0.1 and FDR < 0.05) (Figure 7D).

To further study the dysregulation of glutathione function, the analysis of pathways affecting glutathione metabolism was performed on the RNA-seq data. Our analysis highlighted downregulation of multiple genes involved in glutathione biosynthesis and metabolism alongside alterations of genes of the methionine cycle, transsulfuration, and antioxidant pathways between *Luc* mRNA *Asl^Neo/Neo^* mice and WT livers (Figure 7E). These findings additionally support disruption of glutathione metabolism in *Asl^Neo/Neo^* mice, including the downregulation of *Gclc*. These pathways were corrected post-*hASL* mRNA treatment as shown by post-pathway analysis comparison between *hASL* mRNA- and *Luc* mRNA-treated *Asl^Neo/Neo^* mice (Figure 7E).

NO deficiency is a hallmark of ASA (4, 5). Previous reports have shown that NO donors can upregulate the rate limiting enzyme of glutathione biosynthesis GCL in vascular smooth muscle cells (38) and endothelial cells (39). To test the role of NO on glutathione, we incubated human hepatocellular carcinoma derived Huh7 cells with NO donor S-nitroso-N-acetylpenicillamine (SNAP) versus vehicle (DMSO) control and observed a significant increase in mRNA expression of both GCL subunits, GCLC (Figure 7F) and GCLM (Figure 7G). This suggests that the normalisation of ASL expression corrects hepatic glutathione metabolism by restoring the NO availability and thereby GCL upregulation (Figure S12).

## Discussion

Glutathione is a master antioxidant, a mitochondrial protectant, and regulator of multiple redox processes (40, 41). Glutathione depletion is a well-identified feature of common chronic liver diseases, for example non-alcoholic fatty liver disease, alcoholic liver disease, and cholestasis (42). Generally, raised production of reactive oxygen species causes sustained oxidative stress and scavenges glutathione, although occasionally, compromised glutathione biosynthesis or increased glutathione recycling have been observed (25).

Here we observed glutathione depletion as a key feature of the chronic liver disease in ASA. Glutathione depletion is likely to be multifactorial, with various mechanistic explanations identified in this work, such as decreased biosynthesis, increased degradation, and increased urinary excretion. Although it has been shown that glutathionuria can cause systemic glutathione depletion (43), the normalisation of liver glutathione levels after *hASL* mRNA therapy shows the importance of dysregulated glutathione biosynthesis in the liver. In particular, we observed downregulation of the rate-limiting enzyme of glutathione biosynthesis, GCL, affecting both the catalytic and modifier subunits. The upregulation of the xCT transporter in the liver suggests a feedback mechanism to alleviate the consequences of glutathione depletion. This mechanism has been shown in cancer cells to promote glutathione biosynthesis and thereby cell survival from an increase of the intracellular cysteine pool, and can be observed as well in monogenic liver diseases (21, 44).

These findings contrast with only moderate evidence of oxidative stress and downregulation of genes involved in antioxidant pathways. In ASA livers, the main glutathione-dependent functions and related pathways involved in antioxidant activity and endogenous and xenobiotics detoxification were downregulated. Increased oxidative stress is common in ASA and has been described systemically (5), and in neuronal (9) or endothelial (22) cells. Direct toxicity from argininosuccinate and conjugates (7, 20, 45), and NO deficiency are two pro-oxidant mechanisms. NO deficiency is caused by arginine depletion and subsequent NOS uncoupling (4, 5, 9). NOS uncoupling alters physiological NO synthesis and promotes synthesis of the reactive oxygen species superoxide ion (O_2_^-^). Additionally, at physiological levels, NO can act as a chain-breaking antioxidant capable of attenuating lipid peroxidation (46). In our study however, the *Asl^Neo/Neo^* mouse presents only moderate evidence of hepatic oxidative stress despite evidence of systemic and hepatic NO deficiency.

ASL is the final enzyme required for arginine synthesis in mammals. Arginine is the precursor of NO and consequently, ASA is also a model of inherited NO deficiency. Various clinical symptoms in ASA such as arterial hypertension (5), colitis (47), epilepsy (48), and motor disorder (49) are directly caused by NO deficiency and subsequent downregulation of key physiological processes or metabolic enzymes. NO can upregulate GCL via a non-canonical pathway independent from cGMP in vascular smooth muscle cells (38) and endothelial cells (39). We observed NO-induced *GCL* mRNA upregulation in human hepatocytes, providing a potential mechanistic link between liver NO deficiency and glutathione depletion, however further studies are required to confirm this link. ASS1 and ASL are both part of the same multiprotein complex jointly with NO synthase. ASL plays a key structural role in maintaining this complex (4). Patients with ASLD have a systemic phenotype, presumably caused partly by systemic NO deficiency (15), which patients with ASSD do not have. Various symptoms affecting the ASLD brain, cardiovascular system, gastrointestinal tract, bone physiology have a pathophysiological explanation based on NO deficiency (9, 22, 47–51). Therefore, the clinical, pathophysiological, and molecular evidences of NO deficiency in ASLD are firmly established, whereas evidence of NO deficiency in ASSD remains limited. The potential extrapolation of ASLD glutathione dysregulation in ASSD remains to be proven.

Glutathione dysregulation and ureagenesis defect are two distinct pathways affected by ASA. Ureagenesis can be assessed with multiple biomarkers such as ammonia, amino acids, orotic acid, and stable isotopes, but this does not inform on the status of glutathione biosynthesis and poorly correlates with chronic liver disease (16). To assess glutathione dysregulation, plasma biomarkers could be assessed, such as homocysteine, cysteine, glutathione, glycine, and glutamate. This study, however, highlights the potential of exploiting defective glutathione biosynthesis with non-invasive [^18^F]FSPG-PET imaging as a sensitive diagnostic tool to assess the presence of liver disease in ASA. The use of [^18^F]FSPG-PET imaging is rapidly expanding for clinical cancer imaging (34, 52). Although the use of PET radiotracers in monogenic diseases is a new application, especially in liver indications, we believe this tool will be of interest to academic teams or companies actively developing liver replacement therapies for ASA such as cell or gene (viral (9, 12) or non-viral) therapies. The use of [^18^F]FSPG-PET imaging in assessing the correction of the chronic liver disease in ASA will not be dependent on the therapeutic approach used: a single systemic AAV-mediated gene therapy injection providing long-term phenotypic correction or repeated intravenous LNP-mRNA administrations with sustained efficacy will both be able to benefit from this imaging modality as a non-invasive marker of clinical improvement. The ability to track the effective correction of impaired glutathione metabolism in the ASA liver provides substantial benefits over other more invasive techniques. Dysregulation of glutathione metabolism and antioxidant pathways were fully corrected following mRNA therapy, as observed in liver transcriptomics and liver glutathione concentrations. Liver [^18^F]FSPG retention in neonatally-treated animals was halved compared to untreated mice, however radiotracer retention was not completely normalised to WT levels. This could be due to the experimental design, which included a short 2-week period of therapy to achieve age-matched comparison with untreated control *Asl^Neo/Neo^* animals. We note that the correction was limited to the liver, with no benefit observed in the skin, demonstrating the preferential liver-targeting effect of mRNA therapy as previously described (53).

The lack of effective therapies for both ureagenesis and the chronic liver disease in ASA has promoted the development of various experimental therapies. The autophagy enhancer Tat-Beclin-1 (TB-1) peptide has shown improved ureagenesis along with reduction in hepatocellular injury and glycogen accumulation that may prevent long-term hepatotoxicity (6). Restoration of ureagenesis was achieved using liver-targeting viral-mediated gene therapies using adenoviral (5, 11), adeno-associated viral (AAV) (9, 12), or lentiviral vectors (10). For viral vector-mediated gene delivery, there are ongoing concerns over capsid immunogenicity and toxicity. There have been recent reports of serious hepatotoxicity following AAV gene delivery in X-linked myotubular myopathy and spinal muscular atrophy (54–57). In parallel, mRNA encapsulated in lipid nanoparticles is emerging as a promising therapy for liver monogenic diseases (58–60). This technology enables the delivery of a functional therapeutic protein to target cells with comparatively longer half-life and lower costs than protein replacement therapies. The absence of acute immunogenicity and integration in the host genome enables safe repeating administration to compensate for mRNA degradation and transient efficacy, becoming a viable alternative to viral vectors (61, 62). Proof of concept mRNA therapy in liver inherited metabolic diseases has increased in frequency in recent years (36, 37, 63, 64), supporting data for early phase clinical trials for ornithine transcarbamylase (NCT04442347), propionic acidemia (NCT04899310), methylmalonic acidemia (NCT04159103), and glycogen storage disease type 1A (NTC05095727). Based on previous proof of concept studies performed in another UCD, arginase deficiency (65, 66), we tested a therapeutic dose of 1 mg/kg of *hASL* mRNA delivered weekly through systemic routes of administration in the *Asl^Neo/Neo^* mouse. The treatment of animals from birth showed normalisation of the macroscopic phenotype, plasma metabolites, *in vivo* ureagenesis, and liver ASL expression and activity similar to physiological levels. Comparatively, induction of therapy in early adulthood showed partial but still very effective phenotypic rescue. The observation that arginine concentrations were unable to be rescued, however, remains unexplained. Overall, this study showed proof of concept that mRNA therapy is both safe and efficacious for both early-onset and rescue therapy in a hypomorphic mouse model of ASA with severe phenotype and paves the way for clinical translation. A knock-out mouse model of ASA has previously been described (67) but is no longer available for comparison with our model. Previous translational work in methylmalonic acidaemia has shown similar efficacy of mRNA therapy at same dose and pattern of administration in both severe hypomorphic and knock-out mouse models (63), supporting our findings.

Limitations to our study include the small number of animals per group, which limited the statistical power of some efficacy endpoints. Despite the small sample sizes, however, biochemical and phenotypic differences between WT and *Asl^Neo/Neo^* mice were extensive, as was the rescue effect of mRNA treatment. Small group sizes, however, limited our ability to assess the sex differences of thiol metabolites in patients with urea cycle defects and mRNA-treated *Asl^Neo/Neo^* mice. Additionally, mRNA therapy was studied up to 7 and 9 weeks in neonatally- and adult-treated *Asl^Neo/Neo^* mice, respectively. We cannot exclude that an extended duration of mRNA therapy in ASA might have revealed only partial sustained efficacy due to single organ (liver) correction in this systemic disease. Due to the study design, long-term LNP-related toxicity, a critical aspect for clinical translation, was not studied, although this type of toxicity has not been shown in other LNP-mRNA studies in other murine models of liver inherited metabolic diseases (36, 37).

In conclusion, our study shows dysfunction of glutathione metabolism in both ASL-deficient patients and an *Asl^Neo/Neo^* mouse model, whilst mRNA-LNP therapy corrected both glutathione metabolism and ureagenesis *in vivo*. Preliminary data suggests that glutathione biosynthesis in the liver is regulated by NO availability. Furthermore, we demonstrated the potential of [^18^F]FSPG-PET as a companion diagnostic to assess liver disease and therapeutic efficacy in ASA. These insights into the liver pathophysiology of ASA provide perspectives for targeted therapies, which could change the outcome of patients affected by this rare disease with currently high unmet needs and limited therapeutic options.

## Materials and Methods

### Study design

This study was designed to investigate hepatic glutathione metabolism and its role in the chronic liver disease in ASA. To assess glutathione metabolism in ASA, we first measured thiol metabolites in plasma of ASL-deficient patients. To investigate the cause of dysregulated glutathione metabolism in ASA, we measured plasma and liver thiol metabolites in *Asl^Neo/Neo^* mouse model and performed liver untargeted proteomics. Next, we monitored redox changes in ASL-deficient murine liver using [^18^F]FSPG-PET. We then investigated the therapeutic potential of *hASL* mRNA in correcting dysregulated glutathione metabolism. We first assessed *hASL* mRNA efficacy by ASL supraphysiological overexpression and correction in human derived liver cell line and ASL-deficient fibroblasts, respectively. To evaluate this, we designed pharmacokinetic studies after systemic administration in *Asl^Neo/Neo^* animals of *hASL* mRNA or *Luc* mRNA. Untreated WT littermates were used as controls. *Asl^Neo/Neo^* mice were administered IV at 3-weeks of age and harvested at different timepoints, 2, 24, 72 h or 7 days. We then designed two survival studies for *Asl^Neo/Neo^* animals treated from birth and early adulthood. We initially assessed whether ureagenesis, the main cause of mortality in these *Asl^Neo/Neo^* mice, was corrected. In the first study, neonatal pups at day 1 were administered mRNA systemically weekly up to 7-weeks of age. In the second survival study of animals treated from early adulthood, mice were given weekly IV administration from day 21 onwards up to 9-weeks of age. Efficacy endpoints were survival, growth, fur pattern, plasma ammonia and urea-cycle related amino acids measured in dried blood spot, urine orotic acid, ^13^C ureagenesis, liver western blot, immunostaining and enzyme activity for ASL. All harvests were performed 48 h after the last injection. Mutant mice were assigned randomly to study groups. All animals were monitored and weighted daily. After assessment of the therapeutic effect on ureagenesis, we assessed the therapeutic effect of *hASL* mRNA on dysregulated glutathione metabolism using [^18^F]FSPG-PET and thiol metabolites. Analysis was performed blindly to genotype. We assessed liver transcriptomics in control and treated *Asl^Neo/Neo^* mice. We investigated the expression of glutathione synthetic enzymes in vitro in the presence of NO donor. *n=3* independent experiments were conducted for each *in vitro* experiment of the study. Animal procedures were approved by institutional ethical review and performed per UK home office licenses PP9223137, 70/14300 and PEFC6ABF1.

### Statistical analysis

Data was analysed and represented using Graphpad Prism 9.0 software. Figures shown mean +/- standard deviation. Kaplan-Meier survival curves were analysed using log-rank test. Pairwise comparison of means was performed using Student’s *t* test (two-sided) for comparison of two groups, assuming that the data was normally distributed. Simple linear regression analysis comparing average slopes per group was performed to compare growth curves. Due to early death of *Luc*-mRNA treated *Asl^Neo/Neo^* mice, the analysis comparing this cohort was performed for the duration of the survival of these animals. For treated adult animals, analysis was performed using data from the timepoint of the first injection of LNP-mRNA. One-way ANOVA with Tukey’s post-hoc test comparison or two-way ANOVA with Šídák’s post-hoc test comparison was used to compare more than 2 groups. Log transformation was used to compare groups lacking normal distribution. All statistical results for figures 1-7 are summarised in table S6A-G.

## Supplementary Material

Supplemental Matterial

## Data Availability

All data associated with this study are present in the paper or the Supplementary Materials. Requests for ASL LNP-mRNA should be made directly to Moderna Inc. under a material transfer agreement. Requests for data should be addressed to JB or THW. The transcriptomic dataset is available on NCBI Gene Expression Omnibus, accession number GSE222874.

## One-sentence summary

Argininosuccinic aciduria causes defective glutathione metabolism, which can be monitored by PET and corrected by liver-targeted mRNA therapy in mice.

## Editor’s summary

Genetic argininosuccinate lyase (ASL) deficiency results in excess blood ammonia and downstream neurological and metabolic impairments. Gurung *et al*. report that ASL deficiency in patients also causes dysregulation of glutathione synthesis. Lipid nanoparticle-encapsulated human *ASL* mRNA corrected glutathione and urea metabolism and ameliorated liver disease symptoms in a mouse model of the disease. PET imaging of the mice using further suggested that [^18^F]FSPG, as a readout of glutathione synthesis, may have utility in monitoring the extent of disease and treatment response. – Catherine Charneski
