# Supplementary material for: mRNA therapy corrects defective glutathione metabolism and restores ureagenesis in preclinical argininosuccinic aciduria
Source: Sci Transl Med. Author manuscript; Available in PMC 2024 Jan 17. (PMC7615535; doi:10.1126/scitranslmed.adh1334)
Supplement: Supplemental Matterial [file EMS193274-supplement-Supplemental_Matterial.docx]

**Materials and Methods**

**mRNA formulation**: *hASL* and Luciferase (*Luc*) encoding mRNA encapsulated in Lipid Nanoparticles (LNPs) were provided by Moderna Inc. using their proprietary technology. Codon optimized mRNA encoding *hASL* was synthesized in vitro by T7 RNA polymerase-mediated transcription. The mRNA initiated with a cap, followed by a 5' untranslated region (UTR), an open reading frame (ORF) encoding *hASL*, a 3' UTR and a polyadenylated tail. Uridine was globally replaced with N1-methylpseudouridine, previously described (*68*). For in vivo intravenous delivery, LNP formulations were generated. Briefly, mRNA was mixed with lipids at a molar ratio of 3:1 (mRNA:lipid), previously described (*36*). mRNA-loaded nanoparticles were exchanged into final storage buffer and had particle sizes of 80 – 100 nm, >80% encapsulation of the mRNA by RiboGreen assay, and <10 EU/mL endotoxin concentrations.

**Patient Sample Study:** Patients with ornithine transcarbamylase deficiency (OTCD), argininosuccinate synthase deficiency (ASSD) and argininosuccinate lyase deficiency (ASLD) followed at Great Ormond Street Hospital for Children NHS Foundation Trust, London, UK were asked to join a study with research ethics consent 13/LO/0168 issue by the Health Research Authority. Biological data were analysed anonymously.

**Animals:** Animal procedures were approved by institutional ethical review and performed per UK home office licenses PP9223137, 70/14300 and PEFC6ABF1. *Asl^Neo/Neo^* mice (B6.129S7-*Asl^tm1Brle^*/J) were purchased from Jackson Laboratory (strain #018830), and maintained on standard rodent chow (Harlan 2018, Teklab Diets) with free access to water in a 12-hour light / 12 hours dark environment. WT littermates were used as controls and housed in the same cages. Genotyping was performed using DNA extracted from tail clips as previously described (*9*). In-life blood collection for longitudinal analysis was performed via tail-bleed and terminal blood collection via cardiac puncture, followed by cervical dislocation for harvest. Urine samples were collected longitudinally and post-harvest on Whatman filter paper. Animals were culled either at the stated endpoint or at the occurrence of >15% body weight loss or reaching severity limit.

**Pharmacokinetics and survival studies**: *Asl^Neo/Neo^* animals were given systemic administration of *hASL* mRNA or *Luc* mRNA at dose of 1mg/kg or 2mg/kg for IV and IP injections, respectively. Untreated WT littermates were used as controls. For the pharmacokinetics experiment, *Asl^Neo/Neo^* mice were administered IV via tail-vein at 3-weeks of age and harvested at either 2, 24, 72 h or 7 days. In survival study for animals treated from birth, neonate pups at day 1 were administered mRNA intravenously through the temporal superficial vein, followed by intraperitoneal IP administration at day 7 at dose of 2 mg/kg and IV administration via tail-vein weekly from day 14 onwards up to 7-weeks of age. In survival study of animals treated from early adulthood, mice were given weekly IV administration through tail-vein from day 21 onwards up to 9-weeks of age. All harvests were performed 48 h following the last injection. Mutant mice were assigned randomly to study groups. All animals were monitored and weighted daily. In-life blood collection for longitudinal analysis was performed via tail-bleed and terminal blood collection via cardiac puncture, followed by cervical dislocation for harvest. Urine samples were collected longitudinally and post-harvest on Whatman filter paper. Animals were culled either at the stated endpoint or at the occurrence of >15% body weight loss and/or reaching severity limit.

**[^18^F]FSPG PET imaging:** [^18^F]FSPG was synthesized using a GE FASTlab automated synthesis module and quality control performed as previously reported (*69*). Anaesthetized (1.5-2% isoflurane in O_2_) 2-3 week old *Asl^Neo/Neo^* mice with age-matched WT littermates were imaged after IV injection of 1-3 MBq of radiotracer. For rescue experiment with mRNA therapy, *Asl^Neo/Neo^* mice were given 1 mg/kg IV dose of *hASL* mRNA dose at day 1 of birth followed with weekly 2 mg/kg IP injection. Age-matched control untreated *Asl^Neo/Neo^* and WT littermates were included. For all animals, dynamic PET scans were acquired between 40 and 90 min post-injection on a Mediso NanoScan PET/CT system (1-5 coincidence mode; 3D reconstruction; CT attenuation-corrected; scatter corrected) using a four-bed mouse hotel (Mediso) (*70*). CT images were acquired for anatomical visualization (360 projections; helical acquisition; 55 kVp; 600 ms exposure time). A dynamic iterative reconstruction algorithm, Tera-Tomo 3D (0.4 × 0.4 × 0.4 mm^3^ voxel size), was used with attenuation, scatter, and random coincidences correction. Radiotracer concentration was quantified using VivoQuant software (v 2.5, Invicro Ltd), with volumes of interest drawn manually using the CT image as reference. Data were expressed as percent injected dose per gram of tissue (% ID/g). After [^18^F]FSPG PET, mice were culled by cervical dislocation and liver, skin and other tissue was collected, snap frozen and moved to a -80°C freezer for later *ex vivo* analysis.

**Ammonia and ALT measurement:** To obtain plasma samples, whole blood was collected in EDTA tube (Sarstedt) and centrifuged immediately at 13,000 rpm for 5 min at room temperature. Supernatant was then transferred into a microcentrifuge tube and stored at -80^o^C. Ammonia and ALT reads were obtained from Fujifilm NX600 machine using ammonia and ALT cartridges respectively (Fujifilm) using 10µl plasma volume (diluted vol:vol 1:3 in PBS).

**Amino acid analysis:** Liquid chromatography-Mass spectrometry (LC-MS/MS) was used for amino acid measurements (argininosuccinic acid and L-citrulline) from dried bloodspots as described previously (*6*) using the hydrophilic interaction liquid chromatography (HILIC) separation of metabolites, method adapted from (*71*). Briefly, 40µl of whole blood was spotted on Guthrie blood spot card, dried at room temperature for 24h and stored in -20^o^C in a foil bag with desiccant. 3mm blood spot punch was extracted in 100µl methanol containing stable isotopes (2nmol/l, L-citrulline-d7, CDN isotopes), used as internal standards, for 15 min in sonicating waterbath at room temperature. The supernatant was collected and dried using Eppendorf Concentrator Plus and resuspended in 80µl of 0.05M HCl, topped with 280µl of Solvent A (10mM ammonium formiate+85% Acetonitrile (ACN)+0.15%Formic acid (FA)), centrifuged at 16,000rpm for 5 min and supernatant taken for analysis.

Acquity UltraPure Liquid Chromatography (UPLC)-system (Waters) using Acquity UPLC BEH Amide column (2.1x100mm, 1.7µm particle size) and Van Guard UPLC BEH Amide pre-column (2.1x5mm, 1.7µm particle size) (Waters Limited) was used for amino acid chromatography. The mobile phases were (A) 10mM ammonium formiate in 85% ACN and 0.15% FA and (B) 15mM ammonium formiate containing 0.15% formic acid, pH 3.0. Detection was performed using a tandem mass spectrometer Xevo TQ-S (Waters) using multiple reaction monitoring in positive ion mode. The dwell time was set automatically with MRM-transition of 291.2>70.2, 273.2>70.2 and 176.1>159 respectively for ASA, ASA-anhydrides and L-citrulline. L-Citrulline-d7 (183.15>166.05) was used as internal standard control. Argininosuccinate data were analysed using Masslynx 4.2 software (Micromass UK Ltd) and TargetLynx application manager used for subsequent batch analysis.

**Nitric oxide metabolites:** Plasma samples were pre-treated with N-ethylmaleimide (NEM) and deproteinised by precipitation with methanol (v:v 1:1), followed by centrifugation at 16,000 ×g for 20 min. Liver samples were diluted 1:3 (w/v) with homogenisation solution (10mM PBS supplemented with 10mM NEM and 2.5mM EDTA) and homogenised on ice using an all-glass Kimble tissue grinder in combination with a GlasCol GT Series stirrer (8 up-and down strokes). Tissue homogenates were deproteinized in the same manner as plasma. Deproteinized samples were analysed for nitrate (NO_3_^-^) and nitrite (NO_2_^-^) using a dedicated high-performance liquid chromatography analyser (ENO20, Eicom) as described (*72*).

Tissue homogenates were analysed for content of total nitrosation products (RXNO) by gas-phase chemiluminescence of bound NO following reductive denitrosation. Nitrite was removed from sample aliquots by addition of 10% (v:v) of a reaction solution comprising 5% sulfanilamide in 1 M HCl and reaction for 15 min prior to injection into an acidic triiodide-containing reduction chamber. The amount of NO liberated from low-molecular weight and protein nitroso-species was quantified by a gas-phase chemiluminesence analyser (CLD 77 am sp, EcoPhysics), as previously described (*72*).

**Oxidative stress marker and thiol redox status:** Circulating lipid peroxidation products in plasma were evaluated by measuring the thiobarbituric acid reactive substances (TBARS) essentially as described elsewhere (*73*). Malondialdehyde (MDA), a major breakdown product of the peroxidation of unsaturated fatty acids, in plasma or liver homogenate reacts with thiobarbituric acid, at high temperature and acidic conditions, to form a coloured adduct with maximum absorption at 532 nm. After subtraction of background coloration, the resultant absorbance at 532 nm in the sample is then compared to that of a standard curve of solutions of known concentrations of MDA.

Thiol redox status in plasma and liver homogenates was measured using ultra-high performance liquid chromatography tandem mass spectrometry (UPLC-MS/MS) following derivatization with the thiol alkylans NEM, as described in detail elsewhere (*74*). The LC-MS system was used to separate and quantify the biological aminothiols including total glutathione (GSH and GSSG), cysteine (CyS), cystine (CySS), homocysteine (HCyS), homocystine (HCySS), glutamyl-cysteine, cysteinylglycine as well as sulfide. In addition to the free thiols in the sample, their total concentrations (free + protein-bound forms and disulfides) were determined after sample pre-processing with dithiothreitol (DTT). For this purpose, aliquots of plasma and tissue homogenates already reacted with NEM were subjected to reduction by addition of 50mM DTT (1:1 v:v). After incubation for 30 min at room temperature for complete reduction, excess NEM (100mM; 1:10, v:v) was added for derivatization of the liberated thiols and samples were processed as before. NEM-derivatized sample aliquots were spiked with stable-isotope labelled internal standards, subjected to ultrafiltration for protein removal and diluted in 10 mM ammonium phosphate buffer before analysis by LC-MS/MS.

**ASL enzyme activity:** For liver ASL activity, 20-30mg of liver was homogenised in 400µl of cold homogenising buffer (50mM phosphate buffer pH 7.5 and 1x Roche EDTA-free protease inhibitor (Roche)) using Precellys homogeniser tube (VWR) and Precellys 24 tissue homogeniser (Bertin Instruments), centrifuged at 10000g for 20 min at 4^o^C and protein levels measured from the supernatant using BCA kit (ThermoFisher Scientific). 60µg of protein lysate was incubated with 3.6mM ASA in final volume of 50µl, incubated at 37^o^C for 1h followed by reaction termination at 80^o^C for 20 min. The mixture was centrifuged at 10000g for 5 min and 5µl of the supernatant was used to measure fumarate concentrations per instruction from the commercial fumarate kit (Abcam).

For ASL enzymatic assay in fibroblasts, 800,000 cells were plated 24h before in a 6cm tissue culture grade dish. 2.5µg of hASL mRNA-LNPs or Luc mRNA-LNPs were transfected and incubated for 48h. Cells were harvested in 250µl of assay buffer provided in fumarate kit. 60µg of protein per samples was incubated with 300µM of ASA in final volume of 100µl, incubated for 15 min at 37^o^C followed by reaction termination at 80^o^C for 15 min. Samples were then centrifuged at room temperature for 5 min at maximum speed on benchtop centrifuge. 50µl of supernatant was used for fumarate reaction to determine fumarate reaction per the kit instructions.

**Orotate measurement:** Urine was spotted on a Whatman filter paper and dried over 24h room temperature and stored in -20^o^C in a foil bag with desiccant, method adapted from (*75*). For extraction, a 3mm punch of the urine was eluted in 150µl of ddH_2_O containing 40µM labelled orotic acid (stable isotope 1,3-15N2 orotic acid, Cambridge isotopes) and creatinine-D3 (N-Methyl-D3, CDN isotopes) at room temperature for 3h. Orotic acid was analysed in negative ion mode using LC-MS/MS on waters Xevo-TQ-S with MRM transitions (155.1>111.1 and 157.1>113.1) respectively for labelled and unlabelled orotic acid. An isocratic method was used with sample eluted at flow rate of 0.25ml/min with 40% solvent A (water +0.1% FA) and 60% ACN for 1.5 min followed by wash with 100% ACN for 0.5 min and 0.5 min of initial starting condition. Creatinine was used as control to normalise orotic acid concentrations from the same extracted sample. Creatinine was measured in positive ion mode with MRM transitions (113.95>43.85 and 116.95>46.85) for unlabelled and labelled creatinine respectively with same LC-MS/MS conditions as orotic acid.

**Cell culture:** Huh7 cells were obtained from Creative Bioarray (CSC-C9441L). Fibroblasts cells were maintained in Dulbecco’s modified Eagle medium (ThermoFisher Scientific, 41965062) supplemented with 10% (vol/vol) Fetal Bovine Serum (Sigma-Aldrich, F9665) and 50 units of Penicillin and Streptomycin (ThermoScientific, P4458) and maintained at 37^o^C in a humidified 5% CO_2_-air atmosphere. Healthy control fibroblast line was obtained commercially (Lonza, CC-2511). ASL deficient patient fibroblasts were obtained from 2 patients who joined a study with research ethics consent 13/LO/0171 issue by the Health Research Authority. The genotype of patient 1 was c.437G>A / c.437G>A; R146Q / R146Q. The genotype of patient 2 was c.719-2A>G / c.857A>G; ? / GlN286Arg.

**In-cell western:** Fibroblasts were plated at a density of 5,000 cells per well in a 96-well plate (CellBind 96 well microplates, 66025-626). The next day cells were transfected with 0.2µg of *hASL* mRNA or *Luc* mRNA per well for 24 hours after which the cells were fixed in ice-cold methanol for 15 minutes. Rest of the procedure was performed in room temperature. Following 3 quick washes with 1xPBS, the wells were blocked with Licor blocking buffer (927-40000, Licor) for 90 minutes followed by incubation with Anti-ASL antibody (Abcam) for 2 hours. The wells were washed 3x with 1xPBS, incubated with anti-rabbit secondary (IRDye 800CW Goat anti-Rabbit IgG 1:1000, 926-32210, Licor) and cell dye (CellTag700, 1:1000, 926-41091, Licor) for 1h and washed 3x with 1xPBS for 5 minutes after. Post final wash, PBS was removed, plates dried and read on Licor Odyssey CLx (settings: 4mm focus offset, lowest setting quality, resolution 169µM, 700 and 800 channel). Acquisition and analysis were performed in the Licor ImageStudio Lite software (Licor).

***Ex vivo* total glutathione quantification:** Frozen liver and skin tissue was thawed on ice and 25-50 mg of tissue was added to prechilled Lysing Matrix D tube (MP Biomedicals) containing ice cold 400 µL 1X passive lysis buffer (Promega; E1941). The tissue was then lysed at 4 °C on a Precellys Evolution (Bertin Technologies); samples run for five 30s cycles at 6700 RPM. Lysates were centrifuged at 15,000 × *g* for 10 min at 4°C and the supernatant taken for analysis. Total intracellular glutathione was determined using the luminescent-based GSH/GSSG-Glo Assay Kit (Promega; V6611) according to manufacturer’s instructions in a white 96-well plate prepared with 5 µL of sample supernatant (neat or 1:10 diluted) along with 5 µL of GSH standards (1-100 µM). Results were normalized to protein concentration, determined using the Pierce BCA Protein Assay Kit (ThermoFisher Scientific) as per the manufacturer’s instructions.

**Western blot:** Western blot analysis for was carried out using the iBind Flex system (ThermoFisher Scientific), a previously published method (*33*), for antibody immunoblotting. To produce lysates frozen liver and skin tissue was processed and the protein concentration determined as above, except, the prechilled ice cold 400 µL RIPA buffer, with 1% Proteinase and phosphatase inhibitors (HALT) was used instead of 1X passive lysis buffer.

Membranes were probed using rabbit polyclonal anti-xCT (1:500; Novus Biologicals; NB300-318). Actin was used as a loading control for all experiments (1:1000; Cell Signaling Technology; 4967). HRP-linked anti-rabbit IgG (1:200, Cell Signalling Technology; 7074) was used as secondary antibody.

Protein bands were visualized using ECL Prime Western Blotting Detection Reagent (Cytiva; RPN2236) as per the manufacturer’s instructions and the iBright Imaging System (ThermoFisher Scientific). Image analysis and band quantification was performed using the iBright Analysis Software (ThermoFisher Scientific). xCT protein signal per sample was normalised against actin which was used as a loading control.

For ASL and nitrotyrosine analysis in liver, 30mg of liver was homogenised in ice-cold 1x RIPA buffer (Cell Signalling) using Precellys homogenising tube and homogeniser, centrifuged at 10000g for 20 min at 4^o^C and protein abundance measured from the supernatant using BCA kit. 40µg of protein per sample was diluted 1:1 with 2x Laemmli sample buffer (containing 10% 2-β-mercaptoethanol (β-ME)) at final volume of 40µl, vortexed and heated to 95^o^C for 10 min. SDS-PAGE was used to separate the proteins at 100V for 1h followed by wet transfer of proteins into an immobilin PVDF membrane at 400mA for 1h. The membrane was blocked in 5% non-fat milk powder in PBS-T (1xPBS with 0.1% tween-20) followed by overnight incubation at 4^o^C with primary antibodies (Anti-ASL, Abcam ab97370, 1:1000; Anti-GAPDH mouse, Abcam ab8245, 1:10,000; Anti-nitrotyrosine, Merck 05-233, 1:100; Anti-GAPDH rabbit, Abcam ab9485, 1:1000), 3x 5-min washes with PBS-T, 1h incubation with fluorescent secondary antibodies (IRDye 800CW Goat anti-Rabbit IgG 1:1000, 926-32210 and IRDye 680RD Donkey anti-Mouse IgG, 923-68072, Licor) and 3x 5 min washes with PBS-T. Image acquisition and analysis was performed using Licor Odyssey and image analysed using Licor ImageStudio Lite software. ASL protein signal per sample was normalised against GAPDH which was used as a loading control.

**Histology:** At harvest, liver was fixed in 10% formalin solution, left at room temperature for 48h before transferring and storing in 70% ethanol. The liver was paraffin embedded and sectioned at 5µM thickness. Sections were dewaxed in histoclear, hydrated through graded ethanol solution to water followed by incubated in 1% H_2_O_2_ to remove blood stains. Antigen retrieval was performed in boiling 0.01M citrate buffer for 20 min and then cooled to rt. The slides were blocked in 15% goat-serum and TBST-T for 30 min in rt then incubated overnight with primary antibody 9Anti-ASL, ab97370, Abcam, 1:1000) in 10% goat serum and washed 3x with TBST-T. DAB staining was performed using Polink-2 Plus HRP Polymer and AP Polymer detection for Rb antibody kit (D39-18, Origene) following the manufacturer’s instructions. The slides were then dehydrated with increasing gradient of ethanol to water and histoclear. The slides were mounted using non-aqueous mounting media – Microscopy DPX (Merck) and dried overnight.

The slides were imaged under Zeiss Axioplan Histology scope at UCL Great Ormond Street Institute of Child Health Imaging Facility. Ten images per condition was taken in random and averaged. Images were analysed using Fiji software using macro written by Dr. Dale Moulding from UCL Great Ormond Street Institute of Child Health Imaging Facility. The macro utilises colour deconvolution to quantify DAB percentage coverage to calculate percentage of ASL positive regions.

**Hematoxylin & Eosin (H&E) staining:** The paraffin embedded sections were dewaxed in histoclear solution (Scientific Laboratory Supplies, HS-200) for 5 minutes. Sections were then dehydrated through 3 changes of ethanol (100%, 95% to 75%) for 5 minutes each and rinsed in water for 5 min. This was followed by staining in haematoxylin solution (Vector Laboratories, H3401) for 10 minutes and rinsing in running tap water for at least 5 minutes. Staining in working 0.5 % eosin Y solution (Merck, 109844) for 2 minutes was performed and followed by a water wash for 1 min. Dehydration of the samples was finally done through 3 changes of ethanol (from 75%, 95% to 100% ethanol) for 5 minutes each. Clearing of the samples was done using into histoclear. Last, a drop of DPX mount (Merck, 1.00579.0500) was placed on each tissue and a coverslip added.

**GGT Activity:** GGT activity was measured using colorimetric Abcam kit (ab241029, Abcam). 10-20mg of frozen liver sample was homogenised in 200µl of GGT Assay Buffer provided in the kit, then centrifuged (13,000g for 10 min) to remove insoluble material. 10µl per sample was used for the assay following kit instructions. The GGT activity was normalised to protein abundance per sample.

**^13^C ureagenesis:** 30 min pre-harvest animals were given intraperitoneal (IP) administration of 1% body weight labelled sodium acetate (1,2-^13^C_2_, 99%, CLM-440-1, CK Isotopes). Plasma was harvested as before and stored at -80^o^C until analysis. Samples were processed and analysed using isotope-ratio mass spectrometry. Mouse plasma (25 µL) was deproteinized by addition of 25µL of 60% perchloric acid and 0.5 mL 5mM urea (added as unlabelled carrier), sample vortexed, and centrifuged at 21,130g for 5 minutes to remove precipitated protein. The tube was then left uncapped for 30 minutes at room temperature to facilitate evaporation of CO_2­_. The supernatant was transferred to a new microcentrifuge tube and 100µl 0.5M potassium phosphate added. The pH was adjusted to 4 –7 with 1M KOH solution using pH strips. Sample centrifuged at 21,130g for 5 minutes to remove the precipitated potassium perchlorate. The supernatant was added to an ion exchange column (1ml Dowex-1 1X8-200 resin in empty polypropylene SPE Tube), and eluant collected into a 12ml Exetainer (Labco. UK Ltd). The ion exchange column was washed with 2mL 10 mM HCl, and the eluant combined with the earlier fraction in the Exetainer. Samples were dried under N_2_ at 80°C. Dried samples were left uncapped in sealed dessicator for 18 hours with gauze soaked in 1M NaOH to absorb any residual trace of bicarbonate/CO_2.._ The tube was sealed with a cap, and flushfilled with 75ml/min helium for 5 minutes per tube. Meanwhile, 8ml 0.5 M potassium phosphate, pH 6.0 was heated to boil for 5 minutes to remove dissolved gas, then cooled. After cooling, 60mg urease dissolved in buffer by very gentle vortexing. 400 µL urease in potassium phosphate buffer injected through the septum of each vial using a gastight syringe, avoiding introduction of any air bubbles. Samples incubated for 60 minutes at 25°C and 100µL 20% phosphoric acid through the septum, using a gastight syringe, avoiding introduction of any air bubbles. Samples incubated for a further 60 minutes at 25°C to allow full release of CO_2_. Samples analysed by Thermo-Finnigan DeltaPlus XP Plus isotope ratio-mass spectrometer (ThermoFisher Scientific) with Gasbench sample Introduction unit and CTC GC-PAL autosampler, with 10 technical replicates (final 10 of 15 sample injections of 100µL). Urea production was calculated from the ^13^CO_2_/^12^CO_2_ ratio, taking into account the initial dilution by carrier urea, and the concentration of urea in the plasma samples.

**Bioluminescence imaging:** Animals were anesthetized with isoflurane (Abbott Laboratories), injected intraperitoneally with D-luciferin firefly (15mg/ml in PBS) (L-123-10, Gold Biotechnology) at a dose of 150mg/kg and imaged 5 min later with a cooled charge-coupled device (CCD) camera in the IVIS Spectrum in vivo imaging system (IVIS; PerkinElmer). Grey-scale photographs were acquired with a 24-cm field of view and then a bioluminescence image was obtained using a binning resolution factor of 4, a 1.2/f stop and open filter. Regions of interest (ROIs) were defined manually using a standard area for the mouse liver. Signal intensities were calculated with Living Image software (Perkin Elmer) and expressed as photons per second per cm^2^ per steradian. At each timepoint, bioluminescence imaging was carried out with PBS injected control rodents to establish a median baseline; data points were expressed as fold-change over this internal standard for each individual animal.

**Transcriptomics:** RNA was extracted from liver samples using Qiagen RNeasy kits (74004) following kit instructions. Liver samples from the WT, Luc mRNA and hASL mRNA neonatal treated group of *Asl^Neo/Neo^* mutants were analysed. In all cases, cDNA libraries were prepared using the Kapa mRNA Hyper Prep kit (KapaBiosystem) according to the manufacturer’s instructions, and sequenced on an Illumina NextSeq 1000/2000 to generate ~16 million 50-bp paired end reads per sample (UCL Genomics). Fastp was used for adapter trimming, read filtering and base correction. Processed reads were mapped to the GRCm38 mouse reference genome via STAR using gene annotations from GENODE M29. Normalization and differential gene expression analyses were carried out using the DESeq2 R package (v2.12) (*76*) with differentially expressed genes (DEGs) defined on the basis of a log2-fold change > 0.1 or < -0.1, and an FDR-corrected p-value of < 0.05. Volcano plots were generated to visualize the results of analyses between *Luc* mRNA against WT*, hASL* mRNA against WT, and *hASL* mRNA against *Luc* mRNA. The differential expression of specific genes of interest was plotted in a heatmap with their respective molecular pathways. The data were visualized using ggplot2R R package (v3.3.5) (*77*). The dataset is available on NCBI Gene Expression Omnibus, accession number GSE222874.

**Label Free Proteomics:** Excised mouse liver tissue was homogenised on ice in 500 μl of 50 mM Ambic buffer, 2% ASB-14 using the TissueRuptor mechanical homogeniser (Qiagen). After sonication, protein concentration was assessed using the bicinchoninic acid protein assay kit (ThermoFisher Scientific). 300 μg of protein for each individual sample was aliquoted for individual analyses. samples then underwent acetone precipitation. The protein pellets were subjected to in-solution digestion as described previously (*78*). Briefly samples were resuspended and reduced using dithioerythritol then subsequently carboamindomethylated using iodoacetamide and digested using 1 μg of sequence grade trypsin gold (Promega). Label free proteomic analysis was performed using a nanoAquity coupled to a Synapt-G2-Si mass spectrometer with high-definition ion mobility capability (Waters) as previously described (*78*). Raw data were analysed using Progenesis LC–MS (Nonlinear Dynamics Limited) raw data was processed as described previously with the Uniprot mouse reference proteome database. Peptide search settings were performed at a 1% false discovery rate with fixed modification for carboamidomethylation of cysteines and variable oxidation of methionine. Protein identifications with a confidence score > 20 and more than one unique peptide were exported for further analysis along with P values and fold changes values determined by Progeneis QI.

### **Bioinformatics:** Ingenuity Pathway Analysis software (Qiagen) was used to perform in depth canonical pathway analysis and determine biological functions altered in the datasets.

**RT-PCR of GCL and GS:** Liver samples were stored frozen at −80 °C before RNA extraction with the RNeasy kit (QIAgen) according to the manufacturer’s instructions. cDNA was amplified using High-Capacity RNA-to-cDNA Kit (Applied Biosystems). The GCLC sequence was amplified using the following primers: 5′-ACACCTGGATGATGCCAACGAG-3′ (forward) and 5′-CCTCCATTGGTCGGAACTCTAC-3′ (reverse), the GCLM sequence was amplified using the following primers: 5′-TCCTGCTGTGTGATGCCACCAG-3′ (forward) and 5′-GCTTCCTGGAAACTTGCCTCAG -3′ (reverse) and the GS sequence was amplified using the following primers: 5′-CCAGGAAGTTGCTGTGGTGTAC-3′ (forward) and 5′- GCTGTATGGCAATGTCTGGACAC-3′ (reverse). Amplification was detected and normalised against Titin which was amplified using the following primers: 5′-AAAACGAGCAGTGACGTGAGC-3′ (forward) and 5′-TTCAGTCATGCTGCTAGCGC-3′ (reverse). Amplification reactions were carried out using 5 μl of sample, 2.5 μmol.l−1 of each primer, and SYBR green master mix using the Luna universal qPCR master mix (New England Biolabs) for a 25 μl reaction. The amplification conditions were 95 °C for 10 min followed by 40 cycles of 95°C for 15s, 60°C for 45s. Data were processed with StepOne software (ThermoFisher Scientific).

For the nitric oxide (NO) donor experiment, HuH7 cells were plated at density of 500,000 cells per 6-well TC dish (657 160, Cellstar). The next day cells were treated with NO donor S-nitroso-N-acetylpenicillamine (SNAP) (AB120014, Abcam) at 200µM (dissolved in DMSO (D2438, Sigma-Aldrich)) or vehicle (DMSO) control at the same volume as SNAP for 24 hours. RNA extraction and cDNA synthesis was performed as above. Human GCLC sequence was amplified using the following primers: 5′- GGAAGTGGATGTGGACACCAGA-3′ (forward) and 5′- GCTTGTAGTCAGGATGGTTTGCG-3′ (reverse), the human GCLM sequence was amplified using the following primers: 5′- TCTTGCCTCCTGCTGTGTGATG-3′ (forward) and 5′- TTGGAAACTTGCTTCAGAAAGCAG -3′ (reverse). Amplification was detected and normalised against GAPDH which was amplified using the following primers: 5′- GAAGGTGAAGGTCGGAGTCA-3′ (forward) and 5′- TTGAGGTCAATGAAGGGGTC-3′ (reverse). Amplification reaction was carried out as above.

**Reagents and antibodies**: Full list of antibodies provided in **Table S8**, reagents in **Table S9**, and instruments in **Table S10**.

**Supplementary Figures**

**
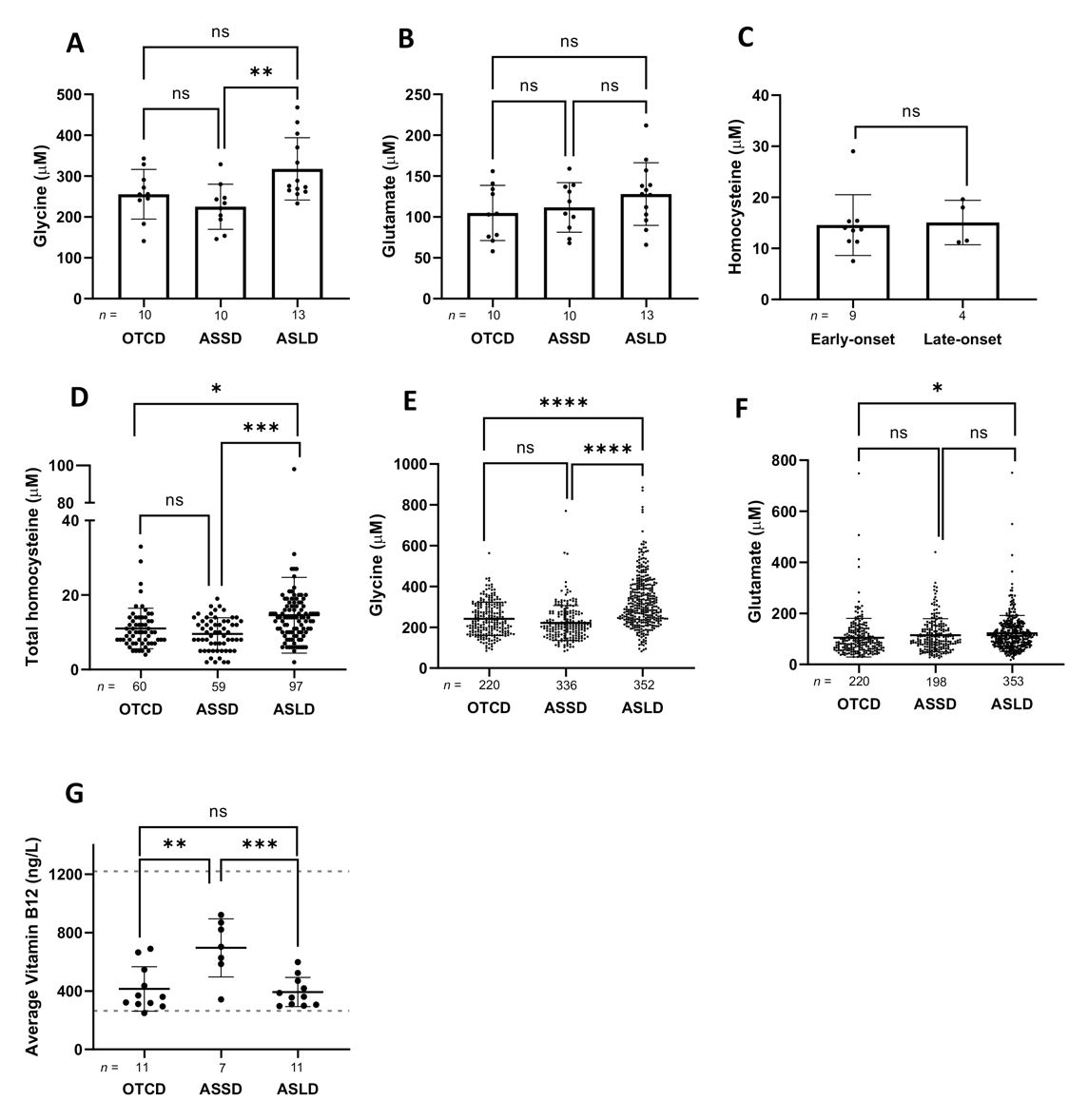
**

**Figure S1. Dysfunction of glutathione metabolism in ASL-deficient patients.**

Mean of plasma (**A**) glutamate and (**B**) glycine in patients with OTCD, ASSD, and ASLD. (**C**) Mean plasma total homocysteine concentrations between early- and late-onset ASLD. Collated single measurements of plasma (**D**) total homocysteine, (**E**) glycine, and (**F**) glutamate (**G**) Plasma vitamin B12 of patients followed for OTCD, ASSD and ASLD, dashed grey lines indicated normal vitamin B12 range. (**A, B, D-F**) One-way ANOVA with Tukey’s post-hoc test (**C**) Unpaired two-tailed Student’s t test; * p<0.05, *** p<0.001, **** p<0.0001, ns not significant. (ASSD: argininosuccinate synthase deficiency; ASLD: argininosuccinate lyase deficiency; OTCD: ornithine transcarbamylase deficiency. Graphs show means $\pm$SD.

**
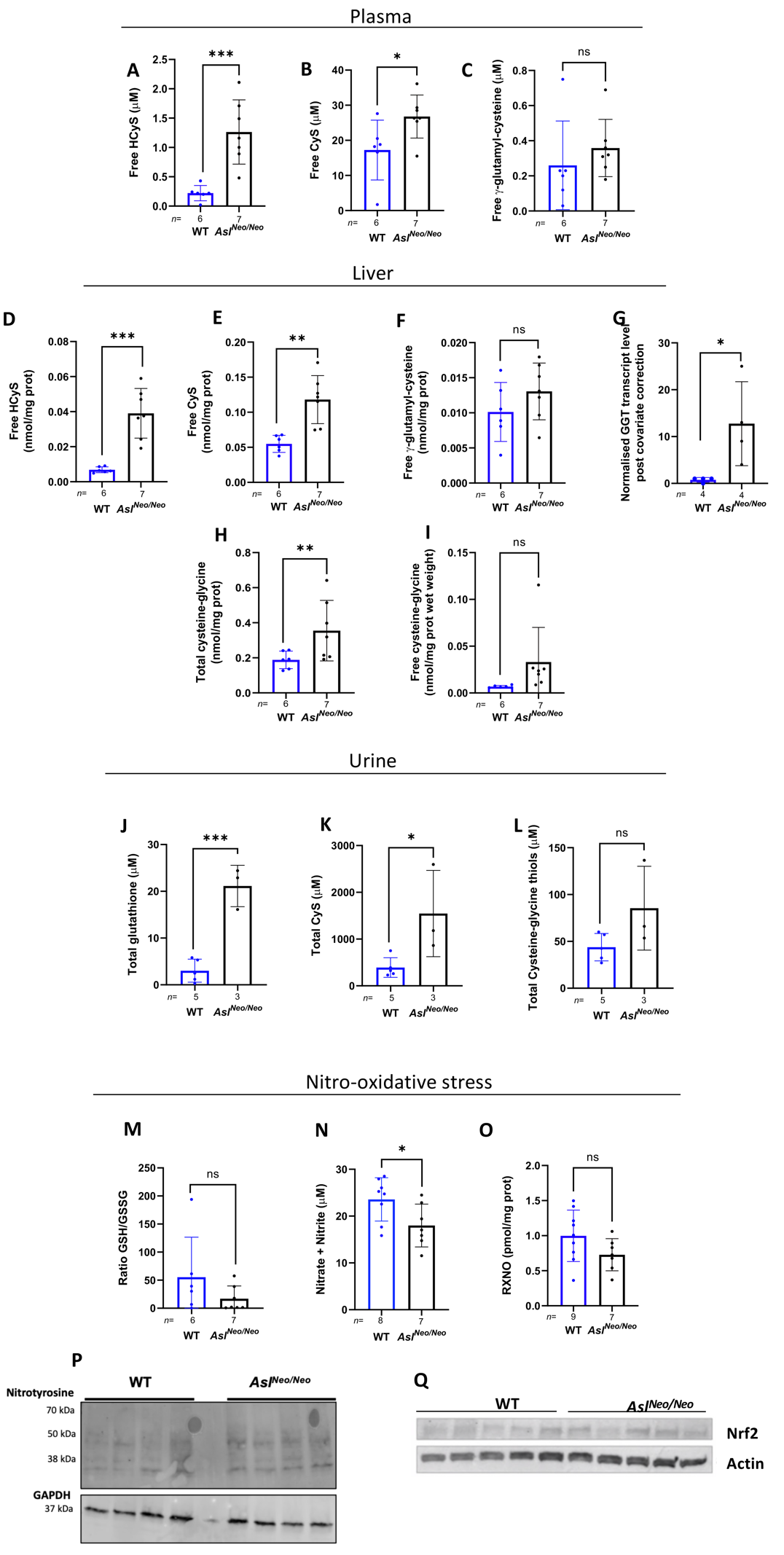
**

**Figure S2. Dysfunction of glutathione metabolism in ASL-deficient mouse model *Asl^Neo/Neo^*.**

(**A**) Free homocysteine, (**B**) free cysteine, and (**C**) free $\gamma$-glutamyl-cysteine in plasma of 2-weeks old *Asl^Neo/Neo^* versus WT littermates. (**D**) Free homocysteine, (**E**) free cysteine, and (**F**) liver free $\gamma$-glutamyl-cysteine in liver of *Asl^Neo/Neo^* mice compared to WT littermates. (**G**) Normalised GGT transcript per million post covariate correction from liver transcriptomics between WT and *Asl^Neo/Neo^* livers. (**H**) Total cysteine-glycine and (**I**) free cysteine-glycine in livers of 2-weeks old *Asl^Neo/Neo^* versus WT littermates. (**J**) Total glutathione, (**K**) cysteine, and (**L**) cysteine-glycine concentrations in the urine of *Asl^Neo/Neo^* mice compared to WT littermates. (**M**) Reduced versus oxidised glutathione ratio in *Asl^Neo/Neo^* livers versus WT littermates. (**N**) Nitric oxide metabolites (nitrite and nitrate) in plasma samples of *Asl^Neo/Neo^* mice and WT littermates. (**O**) Decreased trend of nitroso-species (RXNO), including N-nitrosospecies (RNNO) and S-nitrosospecies (RSNO) in *Asl^Neo/Neo^* livers versus WT littermates. (**P**) Liver nitrotyrosine abundance by western blot between *Asl^Neo/Neo^* mice and WT. (**Q**) Liver Nrf2 abundance by western blot between *Asl^Neo/Neo^* mice and WT. (**A-F, H-O**) Unpaired two-tailed Student’s t test; * p<0.05, ** p<0.01, *** p<0.001, ns not significant. (CySS: cystine; GSH: glutathione; HcyS: homocysteine; HcySS: homocystine; Nrf2: nuclear factor erythroid 2-related factor 2. Graphs show means $\pm$SD.

**
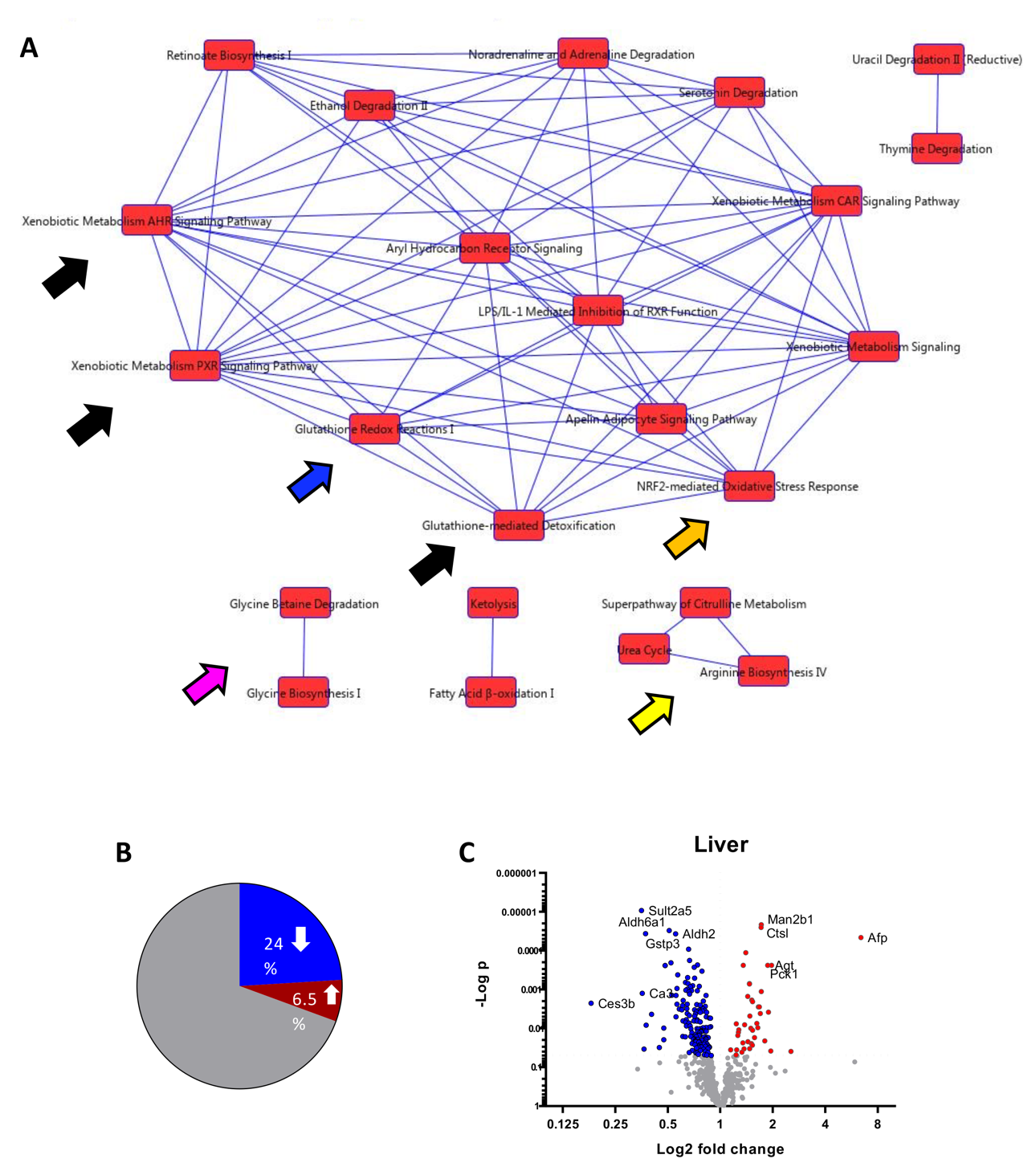
 Figure S3. Dysregulation of liver metabolism in ASL-deficient mouse model *Asl^Neo/Neo^*.**

(**A**) Overlapping canonical pathways showing many major metabolic pathways of the liver were affected: Glutathione antioxidant activity (blue arrow), antioxidant pathways (orange arrow), glutathione-mediated detoxification of endogenous compounds and xenobiotics (black arrows), urea cycle dysregulation (yellow arrow), dysregulation of glycine metabolism (purple arrow). (**B**) Pie chart indicating the percentage effect of the *Asl^Neo^* allele on the mouse liver tissue proteome with majority of significant protein downregulated. (**C**) Volcano plot of differentially expressed proteins in the mutant liver proteome. Significance by FDR adjusted *p* value ANOVA WT n=4, *Asl^Neo/Neo^* n= 5.

**
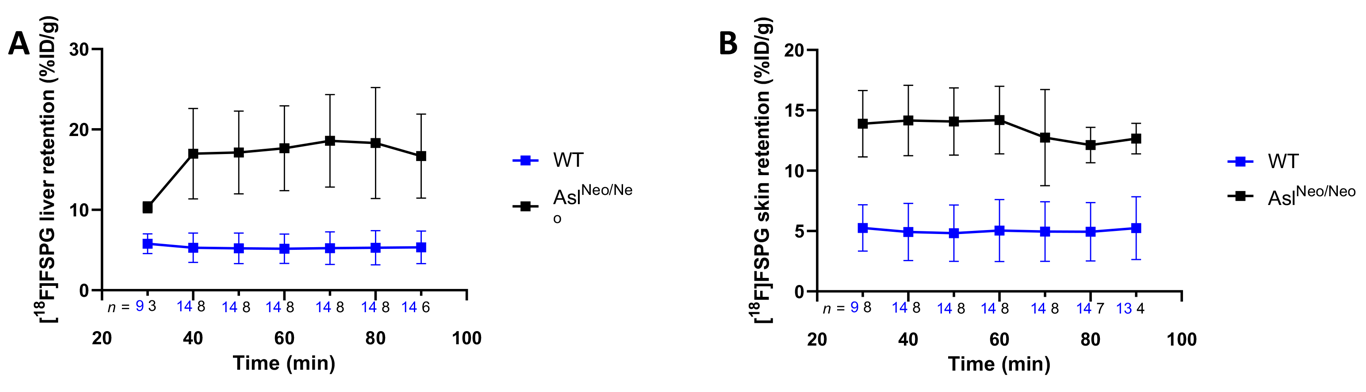
**

**Figure S4. [^18^F] FSPG radiotracer PET scan in *Asl^Neo/Neo^* mice.**

Quantification of [^18^F]FSPG PET retention (%ID/g) in Asl^Neo/Neo^ and WT mice 30 to 90 minutes post-injection in **(A)** liver and **(B)** skin.

**
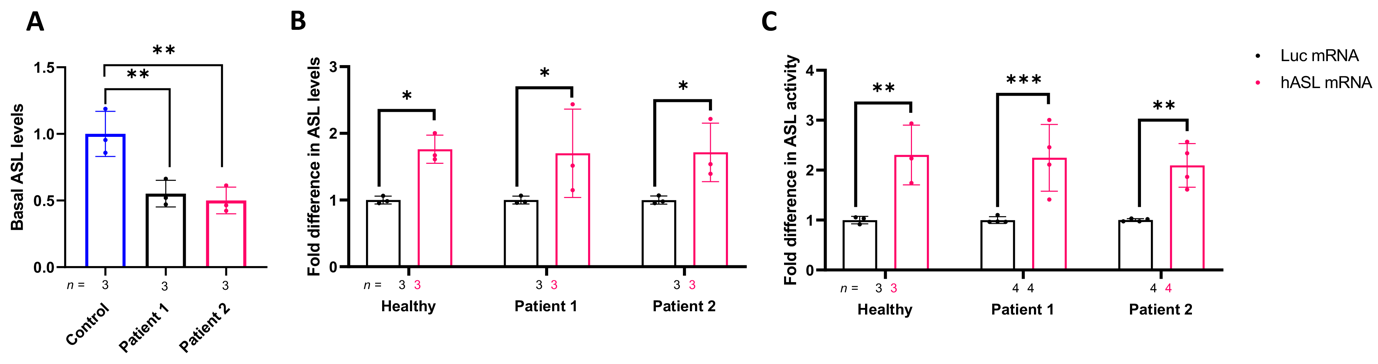
 Figure S5. *In vitro* efficacy of *hASL* mRNA.**

(**A**) Basal expression of ASL in fibroblasts from control and two patients with ASA. Fold difference in (**B**) ASL abundance and (**C**) ASL activity after 24 hours and 48 hours incubation with either *Luc* mRNA or *hASL* mRNA, respectively, from 3 independent experiments and normalised to healthy control (A) and *Luc* mRNA-treated control (B, C). Statistical analysis by One-way ANOVA with Dunnett’s multiple comparisons test against healthy control (A) and two-way ANOVA with uncorrected Fisher’s Least Significant Difference (LSD) (B, C), * p<0.05, ** p<0.01, *** p<0.001. Graphs show means $\pm$SD.

*
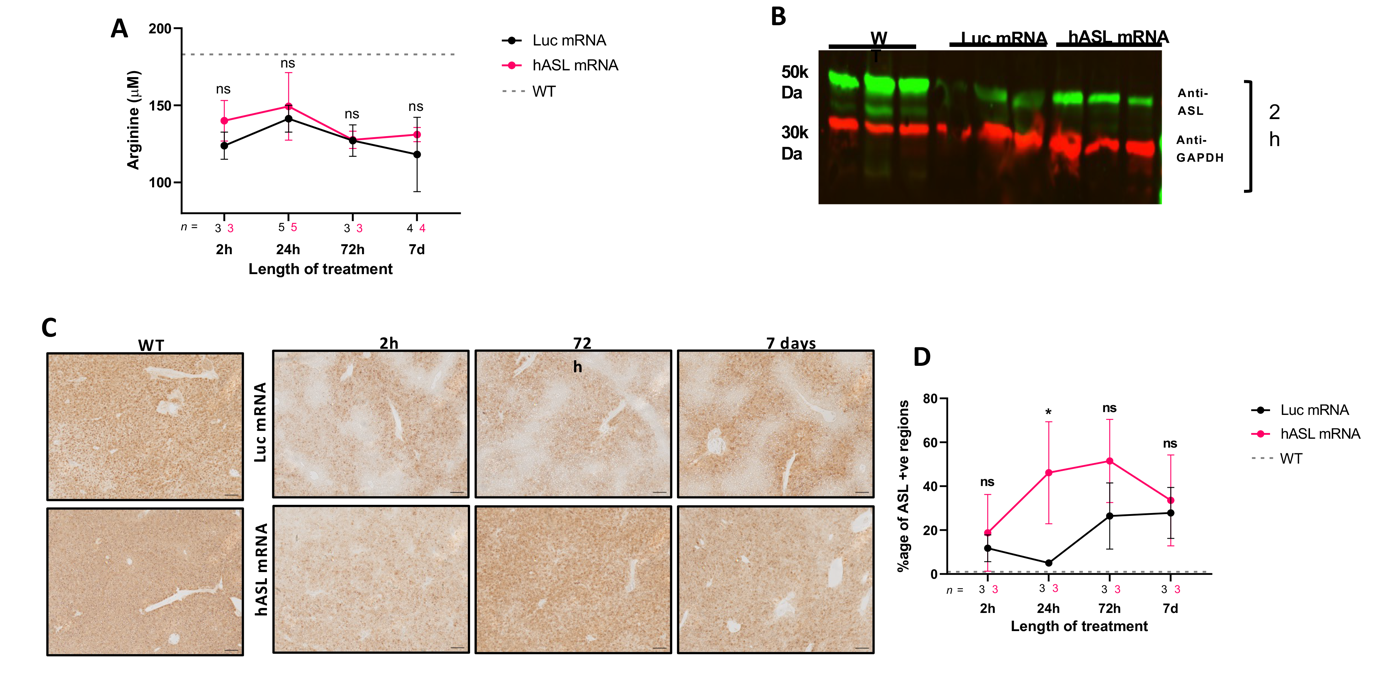
*

**Figure S6. Pharmacokinetics of *hASL* mRNA in *Asl^Neo/Neo^* mice**

(**A**) Average arginine concentrations from plasma from dried blood spots at 2, 24, 72 hours, and 7 days. (**B**) ASL western blot at 2 hours after mRNA administration (n=3). (**C**) Representative images of liver ASL immunostaining at 2 hours, 72 hours and 7 days after mRNA administration from WT and *Luc* mRNA or *hASL* mRNA treated *Asl^Neo/Neo^* mice and (**D**) quantification normalised to WT scaled to 1 (grey dotted line). (**A**) Grey dotted line represents mean WT values (**C**) Scale bar= 100µM. (**A, D**) Two-way ANOVA with Šídák's post-hoc test per group and timepoint, ns=not significant, *p<0.05. Graph show means $\pm$SD.

**
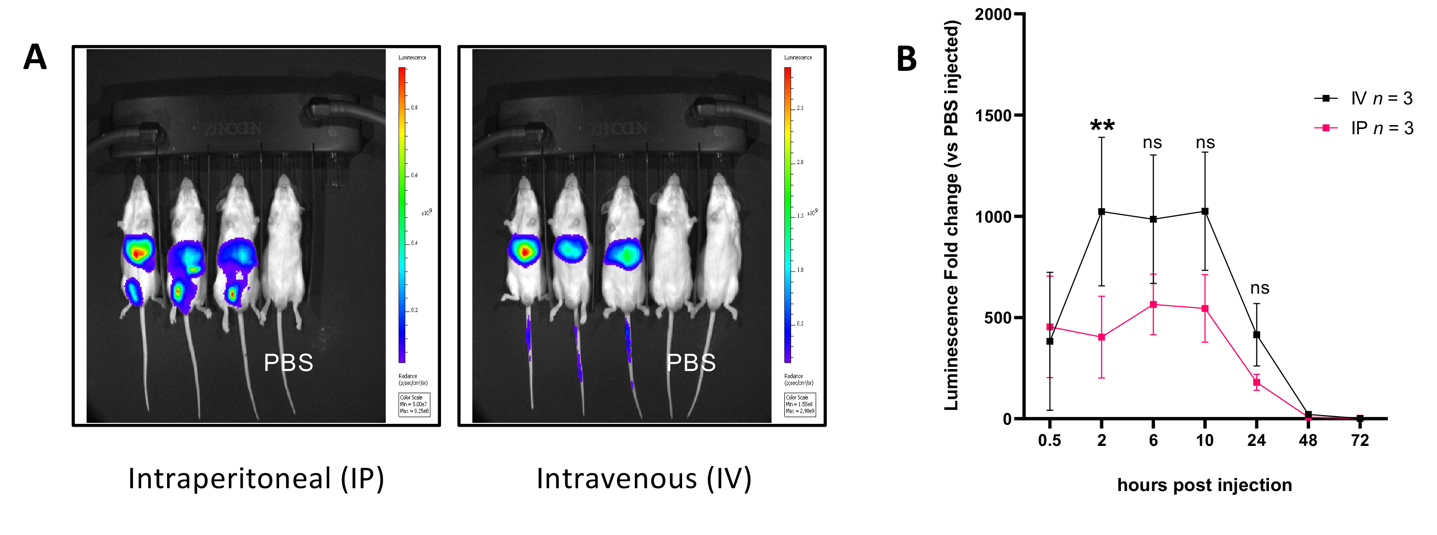
**

**Figure S7. Equivalence of liver biodistribution between intravenous and intraperitoneal administration**

**(A**) Representative *in vivo* luminescence image of WT CD1 strain animals injected with either PBS (IP) or *Luc* mRNA either intraperitoneally or intravenously 10 hours after injection. (**B**) Luminescence fold-change in liver 0.5, 2, 6, 10-, 24-, 48- and 72-hours after injection show two-fold difference in efficacy between IV vs IP. Readings normalised to PBS-injected controls. Two-way ANOVA with Šídák's post-hoc test per timepoint, ns= not significant, **p<0.01, IV and IP n=3, PBS n=4. Graph show means $\pm$SD. IV = intravenous, IP= intraperitoneal.

**
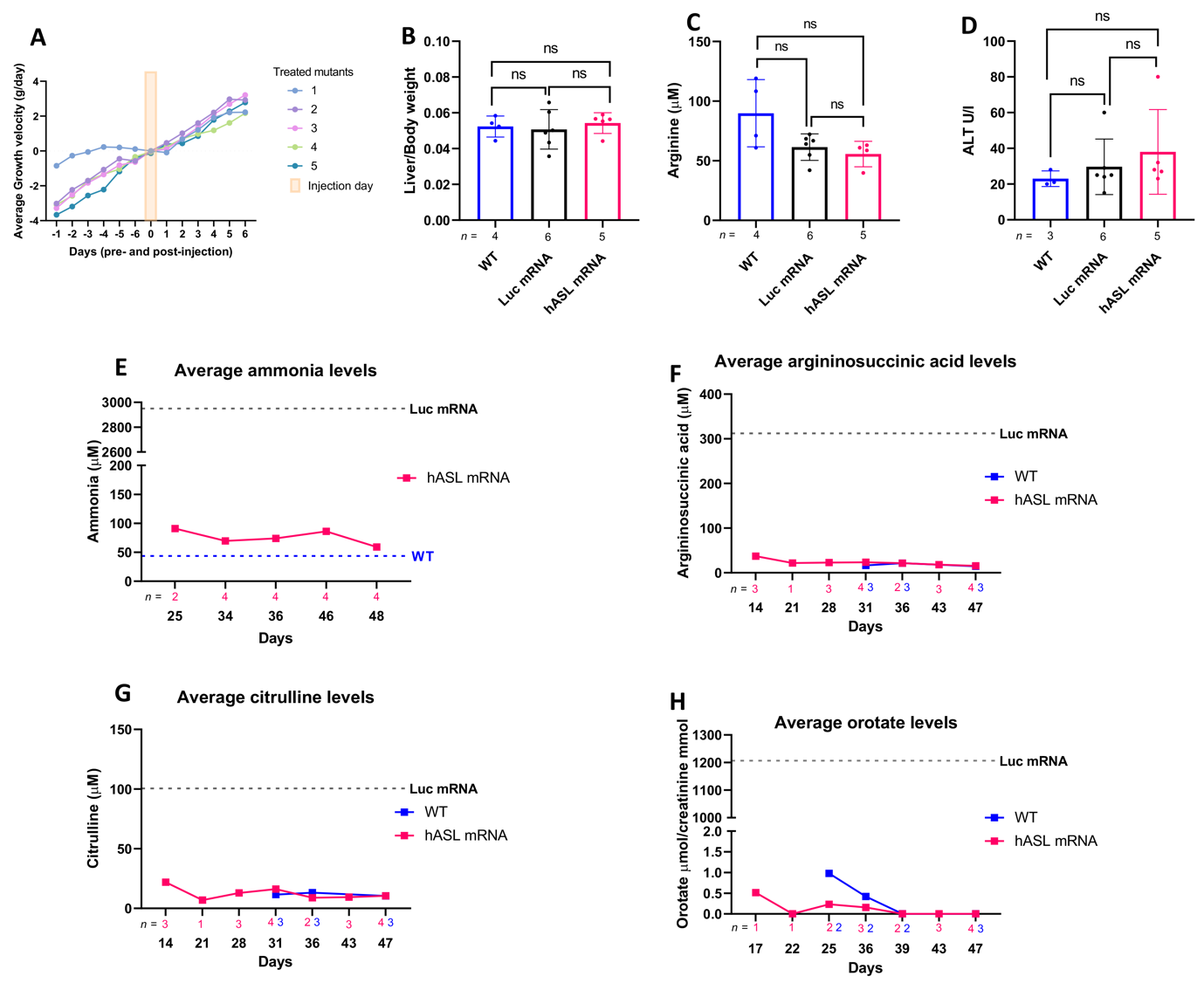
**

**Figure S8. *hASL* mRNA therapy from birth corrects the phenotype of *Asl^Neo/Neo^* mice**

(**A**) Average growth velocity per *hASL*-treated animals each day pre- and post-injection. (**B**) Liver to body weight ratio at harvest comparing WT against *hASL* mRNA treated *Asl^Neo/Neo^* mice. (**C**) Plasma arginine concentrations at harvest from dried blood spots comparing WT against Luc and *hASL* mRNA treated *Asl^Neo/Neo^* mice. (**D**) Plasma alanine aminotransferase (ALT) concentrations at harvest comparing WT against *hASL* mRNA-treated *Asl^Neo/Neo^* mice. Longitudinal (**E**) average plasma ammonia concentration, (**F**) argininosuccinic acid, and (**G)** citrulline from dried blood spots as well as (H) urinary orotate from *hASL* mRNA-treated *Asl^Neo/Neo^* mice. (**B-D**) Unpaired two-tailed t-test per timepoint, ns= not significant. (**E-H**) Grey line indicates average concentration from *Luc* mRNA treated *Asl^Neo/Neo^* mice at harvest (**F**), blue line indicates average WT concentration at harvest. (**A**) Graph shows mean per animal. (**B-D**) Graph show means $\pm$SD. (E-H) Graphs show mean per timepoint.

**
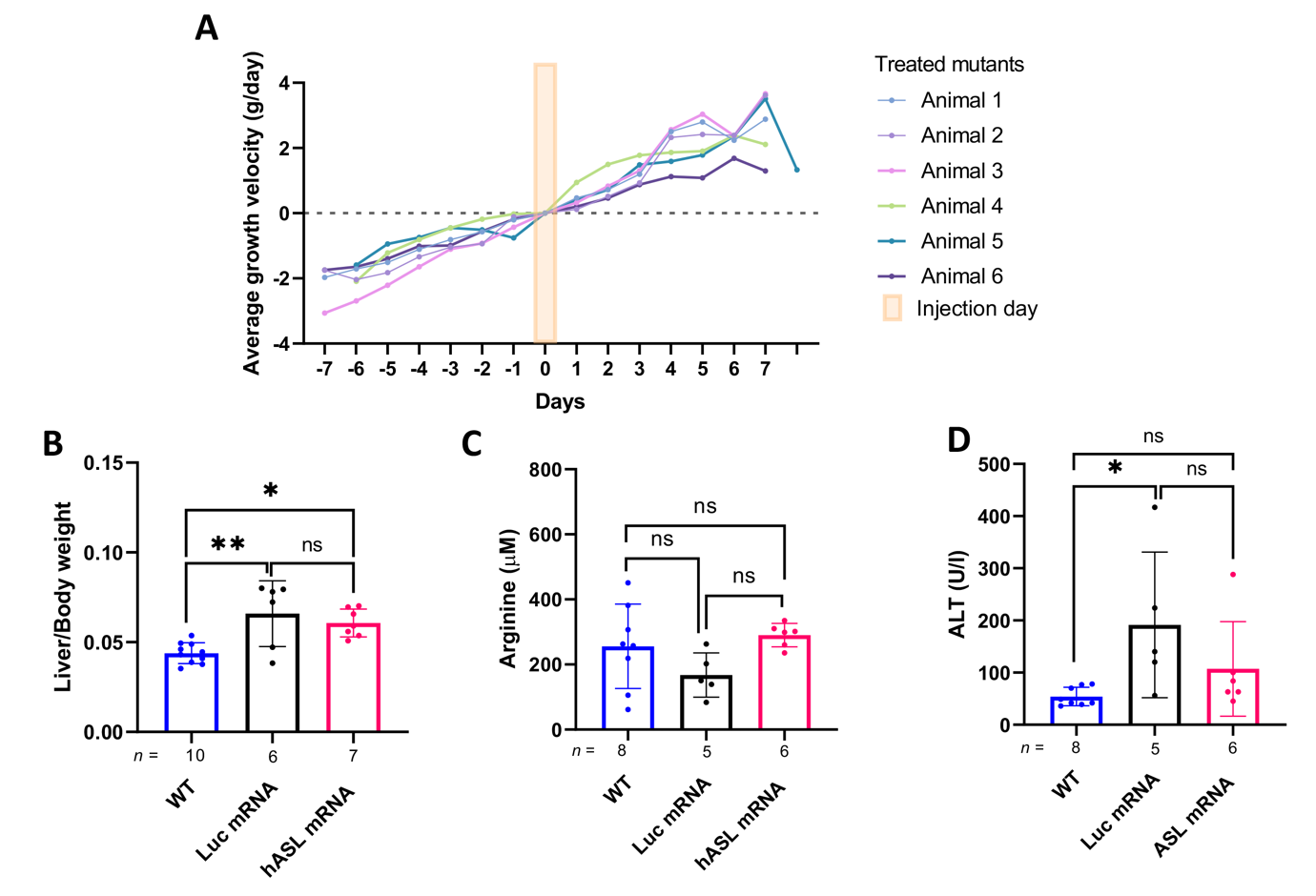
**

**Figure S9. *hASL* mRNA therapy partially corrects the adult phenotype in *Asl^Neo/Neo^* mice**

**(A**) Average mean growth velocity per *hASL*-treated animals each week pre- and post-injection. (**B**) Liver to body weight ratio at harvest comparing *Luc* mRNA against *hASL* mRNA-treated *Asl^Neo/Neo^* mice. (**C**) Plasma arginine concentrations at harvest from dried blood spots comparing WT against *Luc* and *hASL* mRNA-treated *Asl^Neo/Neo^* mice. (**D**) Plasma alanine aminotransferase (ALT) concentrations at harvest comparing WT against *hASL* mRNA treated *Asl^Neo/Neo^* mice. (A) Graph shows mean per animal. (B-D) One-way ANOVA with Tukey’s post-hoc test, ns=not significant, *p<0.05, **p<0.01.


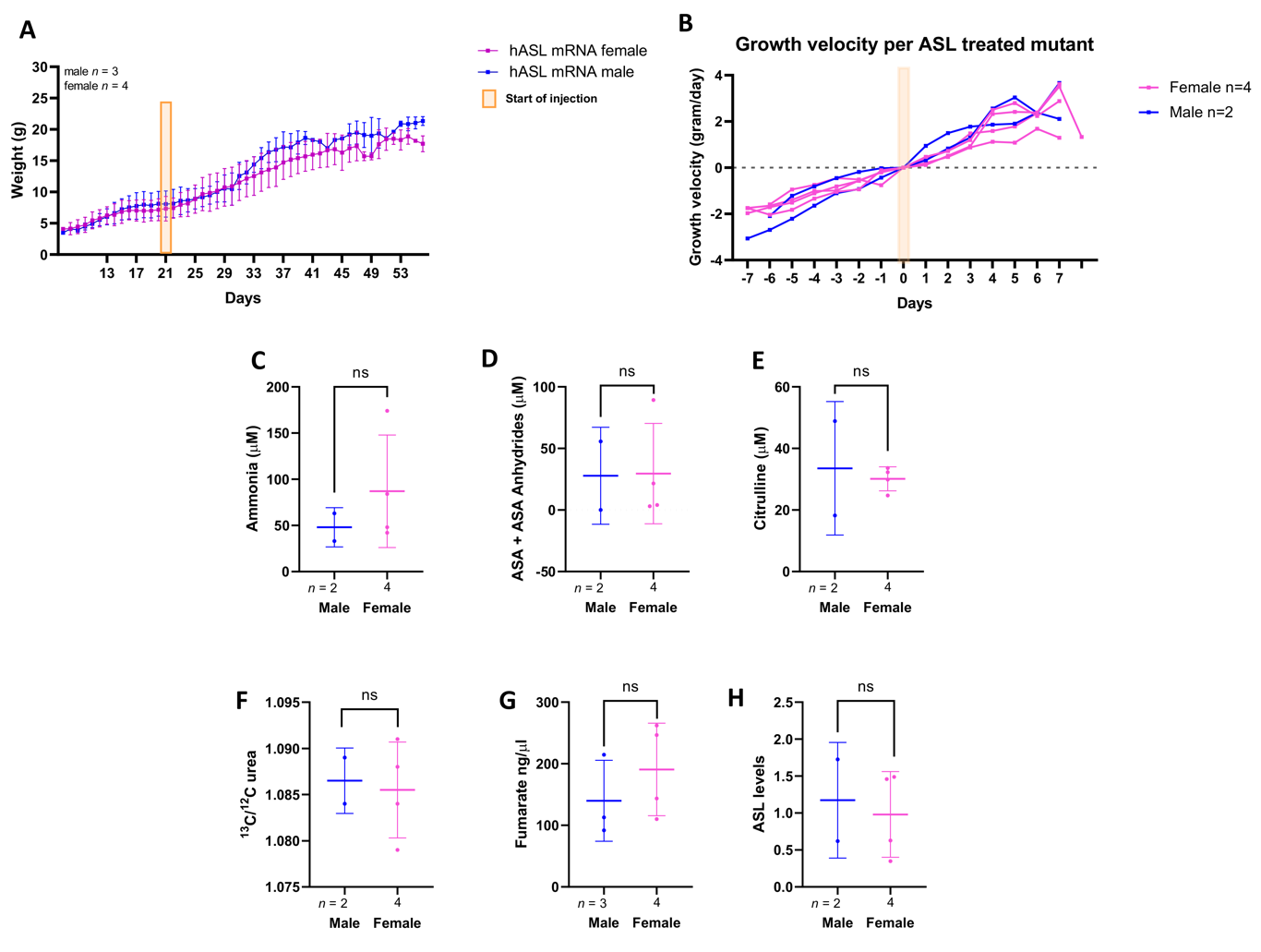


**Figure S10. Gender effect comparison of *hASL* mRNA treatment in adult treated *Asl^Neo/Neo^* mice.**

**(A**) Average growth curve of adult *hASL*-mRNA treated *Asl^Neo/Neo^* mice separated by sex. (**B**) Individual growth velocity per *hASL* treated animals each week pre- and post-injection (pink) female, (blue) male. Average (**C**) plasma ammonia concentrations of *hASL*-mRNA treated *Asl^Neo/Neo^* mice separated by sex. Average (**D**) ASA (**E**) citrulline concentrations from dried blood spots (**F**) C13 ureagenesis from adult *hASL*-mRNA treated *Asl^Neo/Neo^* mice separated by sex. Average liver (**G**) ASL activity indicated by fumarate and (**H**) ASL abundance from western blot analysis of *hASL*-mRNA treated *Asl^Neo/Neo^* mice separated by sex. (**B**) Graph shows mean per animal. (**C-H**) Unpaired two-tailed t-test per timepoint, ns= not significant. ASA: argininosuccinic acid. ASL: argininosuccinate lyase. Graph shows mean $\pm$SD.

**
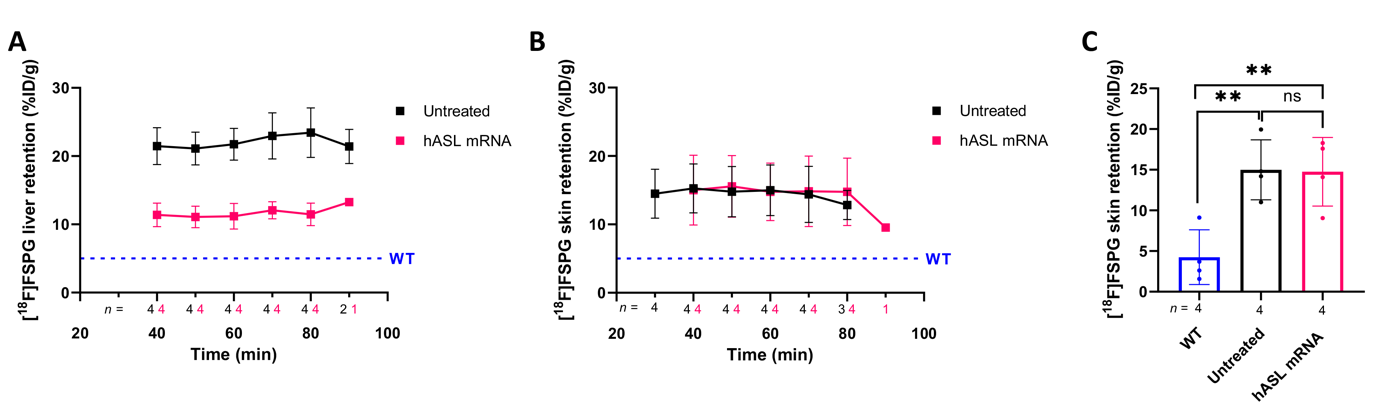
**

**Figure S11. *hASL* mRNA therapy corrects the dysfunction of glutathione metabolism in *Asl^Neo/Neo^* mice**

Quantification of [^18^F]FSPG PET retention (%ID/g) in untreated *Asl^Neo/Neo^* and *hASL*-mRNA treated *Asl^Neo/Neo^* mice 30 to 90 minutes post-injection in **(A)** liver and **(B)** skin. (**C**) [^18^F]FSPG retention in untreated *Asl^Neo/Neo^* and *hASL*-mRNA treated *Asl^Neo/Neo^* mice at 60 min post-injection was threefold higher than that of WT mice. One-way ANOVA with Tukey’s post-hoc test, ns=not significant, **p<0.01.


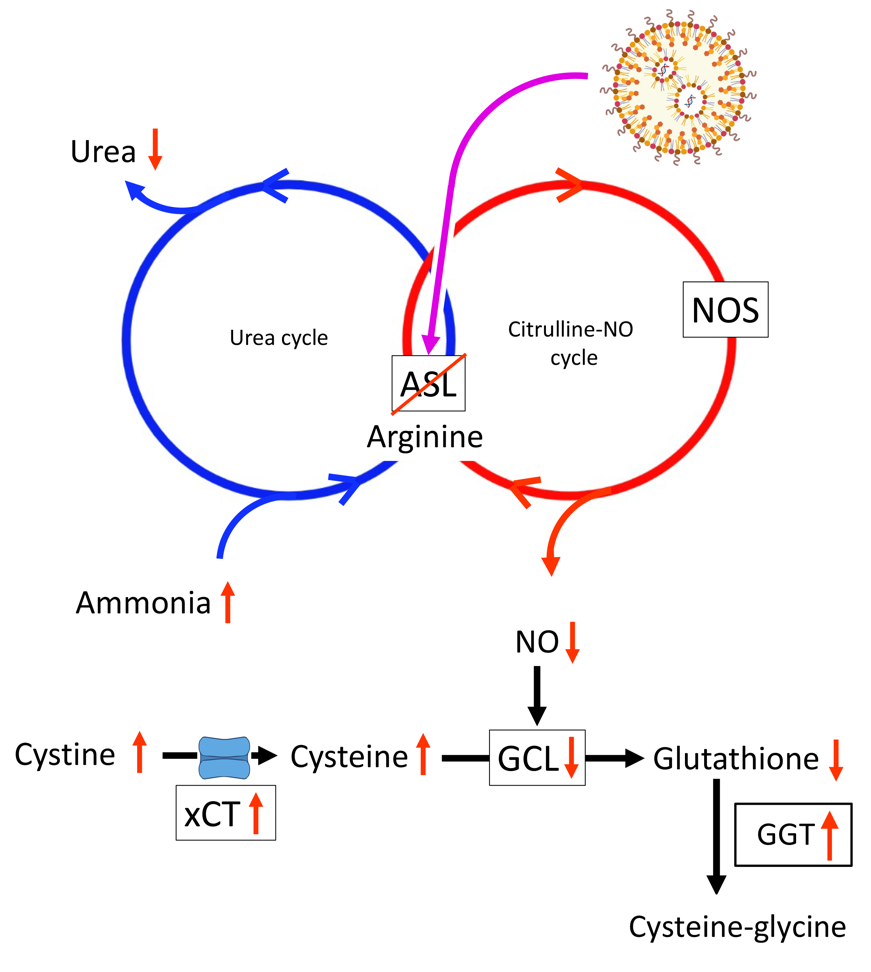


**Figure S12. Schematic highlighting the ureagenesis defect, NO deficiency, subsequent downregulation of glutathione biosynthesis and increased glutathione recycling in the liver caused by ASL deficiency.** All these pathways are corrected by mRNA therapy.

**Supplementary Tables**

**Table S1: Sex analysis of plasma homocysteine, glutamate and glycine concentrations in patients with urea cycle deficiencies** (OTCD n=10, ASSD n=10, ASLD; n=13). ASLD: argininosuccinate lyase deficiency, ASSD: argininosuccinate synthase deficiency, OTCD: ornithine transcarbamylase deficiency. 2-way ANOVA with Šídák's post-hoc test: significance: *p*<0.05.

**Table S2: Information about urea cycle deficient patients regarding genotype, phenotype, therapeutic management, and metabolite concentrations.** ASLD n=13, ASSD n=10, OTCD n=10. ASLD: argininosuccinate lyase deficiency, ASSD: argininosuccinate synthase deficiency, N: normal range values for biological parameters, OTCD: ornithine transcarbamylase deficiency, RFLP: Restriction fragment length polymorphism. (*) Reduced ASS1 activity in fibroblasts 8.7 patom/6h/mg prot (N: 511-5,632)

| Metabolite | **Ammonia** | | | **ASA** | | |
| --- | --- | --- | --- | --- | --- | --- |
| Data analysed | Average | STD | n | Average | STD | n |
| Male | 63.5 | 29.5 | 4 | 15.7 | 1.1 | 4 |
| Female | 41 | n/a | 1 | 15.7 | n/a | 1 |
|  |  |  |  |  |  |  |
| Metabolite | **Citrulline** | | | **C13** | | |
| Data analysed | Average | STD | n | Average | STD | n |
| Male | 9.8 | 2.2 | 4 | 1.07 | 0.002 | 3 |
| Female | 12.4 | n/a | 1 | 1.075 | n/a | 1 |
|  | | | | | | |
| Metabolite | **Orotate** |  |  | **ASL levels** | | |
| Data analysed | Average | STD | n | Average | STD | n |
| Male | 0.0006 | 0.0002 | 4 | 91.4 | 0.9 | 4 |
| Female | 0.0004 | n/a | 1 | 89.8 | n/a | 1 |
|  |  |  |  |  |  |  |
| Metabolite | **ASL activity** | | | **Liver to body weight** | |  |
| Data analysed | Average | STD | n | Average | STD | n |
| Male | 310.2 | 33.8 | 4 | 0.05 | 0.006 | 4 |
| Female | 310.2 | n/a | 1 | 0.06 | n/a | 1 |
|  |  |  |  |  |  |  |
| Metabolite | **ALT** |  |  | **Arginine** |  |  |
| Data analysed | Average | STD | n | Average | STD | n |
| Male | 40.7 | 26.4 | 4 | 129 | 150.7 | 4 |
| Female | 27 | n/a | 1 | 60.9 | n/a | 1 |

**Table S3: Gender effect analysis on neonatal *hASL* mRNA treated *Asl^Neo/Neo^* mice.**

| Metabolite | **Ammonia** | | | **ASA** | | |
| --- | --- | --- | --- | --- | --- | --- |
| Data analysed | Average | STD | n | Average | STD | n |
| Male | 48 | 21.2 | 2 | 27.8 | 39.4 | 2 |
| Female | 87 | 60.9 | 4 | 29.5 | 40.78 | 4 |
| *p* value | 0.31 | | | 0.96 | | |
|  |  |  |  |  |  |  |
| Metabolite | **Citrulline** | | | **C13** | | |
| Data analysed | Average | STD | n | Average | STD | n |
| Male | 33.5 | 21.7 | 2 | 1.08 | 0.0035 | 2 |
| Female | 30.1 | 3.9 | 4 | 1.08 | 0.0052 | 4 |
| *p* value | 0.86 | | | 0.79 | | |
|  |  |  |  |  |  |  |
| Metabolite | **Orotate** |  |  | **ASL levels** | | |
| Data analysed | Average | STD | n | Average | STD | n |
| Male | 8.7 | 11.5 | 2 | 1.17 | 0.78 | 2 |
| Female | 0.45 | 0.53 | 4 | 0.98 | 0.580 | 4 |
| *p* value | 0.49 | | | 0.79 | | |
|  |  |  |  |  |  |  |
|  | **ASL activity** | | | **Arginine** |  |  |
| Data analysed | Average | STD | n | Average | STD | n |
| Male | 139.75 | 65.75 | 3 | 268.7 | 46.8 | 2 |
| Female | 190.67 | 75.12 | 4 | 301.1 | 30.9 | 4 |
| *p* value | 0.38 | | | 0.47 | | |
|  |  |  |  |  |  |  |
|  | **Liver to body weight** | |  | **ALT** | | |
| Data analysed | Average | STD | n | Average | STD | n |
| Male | 0.063 | 0.011 | 3 | 45 | n/a | 1 |
| Female | 0.058 | 0.0052 | 4 | 133.75 | 103.94 | 4 |
| *p* value | 0.53 | | | n/a | | |

**Table S4: Gender effect analysis on adult *hASL* mRNA-treated *Asl^Neo/Neo^* mice.**

**Table S5: Significantly differentially expressed genes in WT vs neonatally treated *hASL* mRNA treated *Asl^Neo/Neo^* mice liver**

Significance: FDR-corrected p-value of < 0.05. TPM: transcripts per million

**Table S6A: Descriptive summary of statistical results for Figure 1.**

| **Figure 1** | | | | | | | | |
| --- | --- | --- | --- | --- | --- | --- | --- | --- |
| **Patient plasma** | | | | | | | | |
|  | **Panel B** | | |  |  |  |  |  |
| Metabolite | **Total homocysteine** | | |  |  |  |  |  |
| Data Analysed | **OTCD** | **ASSD** | **ASLD** |  |  |  |  |  |
| Mean | 9.8 | 9.4 | 14.9 |  |  |  |  |  |
| Median | 9 | 9.7 | 14.1 |  |  |  |  |  |
| SD | 3.9 | 2.6 | 5.5 |  |  |  |  |  |
| SEM | 1.2 | 0.9 | 1.6 |  |  |  |  |  |
| Lower 95% CI | 7.2 | 7.4 | 11.4 |  |  |  |  |  |
| Upper 95% CI | 12.5 | 11.4 | 18.4 |  |  |  |  |  |
| n number | 11 | 9 | 13 |  |  |  |  |  |
| *P* value | | | |  |  |  |  |  |
| OTCD vs ASSD | 0.9 | | |  |  |  |  |  |
| OTCD vs ASLD | 0.02 | | |  |  |  |  |  |
| ASSD vs ASLD | 0.02 | | |  |  |  |  |  |
| **Plasma (mice)** | | | | | | | | |
|  | **Panel C** | | **Panel D** | | **Panel E** | | **Panel F** | |
| Metabolite | **Total homocysteine (mice) µM** | | **Total Cysteine µM** | | **Total glutamyl cysteine** | | **Total glutathione** | |
| Data Analysed | **WT** | **Asl^Neo/Neo^** | **WT** | **Asl^Neo/Neo^** | **WT** | **Asl^Neo/Neo^** | **WT** | **Asl^Neo/Neo^** |
| Mean | 0.2 | 1.2 | 161.3 | 224.9 | 4.4 | 9.2 | 106.4 | 84.5 |
| Median | 0.2 | 1.1 | 151 | 231 | 4.9 | 8.8 | 103 | 85.6 |
| SD | 0.1 | 0.5 | 24.9 | 18.3 | 1.8 | 4.8 | 17.7 | 16.1 |
| SEM | 0.05 | 0.2 | 10.2 | 6.9 | 0.7 | 1.8 | 7.2 | 6.1 |
| Lower 95% CI of mean | 0.08 | 0.7 | 135.1 | 208 | 2.5 | 4.7 | 87.8 | 69.7 |
| Upper 95% CI of mean | 0.3 | 1.7 | 187.5 | 241.8 | 6.3 | 13.7 | 125 | 99.4 |
| n number | 6 | 7 | 6 | 7 | 6 | 7 | 6 | 7 |
| *P* value | 0.0002 | | 0.0003 | | 0.1248 | | 0.0395 | |
| **Liver (mice)** | | | | | | | | |
|  | **Panel G** | | **Panel H** | | **Panel I** | | **Panel J** | |
| Metabolite | **Total homocysteine** | | **Total cysteine** | | **Total glutamyl cysteine** | | **Total glutathione** | |
| Data Analysed | **WT** | **Asl^Neo/Neo^** | **WT** | **Asl^Neo/Neo^** | **WT** | **Asl^Neo/Neo^** | **WT** | **Asl^Neo/Neo^** |
| Mean | 0.05 | 0.1 | 0.4 | 0.7 | 0.05 | 0.09 | 0.2 | 0.07 |
| Median | 0.05 | 0.1 | 0.4 | 0.6 | 0.04 | 0.09 | 0.2 | 0.06 |
| SD | 0.02 | 0.04 | 0.07 | 0.2 | 0.02 | 0.03 | 0.06 | 0.03 |
| SEM | 0.007 | 0.02 | 0.03 | 0.06 | 0.008 | 0.01 | 0.02 | 0.01 |
| Lower 95% CI of mean | 0.03 | 0.1 | 0.3 | 0.5 | 0.03 | 0.06 | 0.1 | 0.05 |
| Upper 95% CI of mean | 0.07 | 0.2 | 0.4 | 0.8 | 0.07 | 0.1 | 0.2 | 0.1 |
| n number | 6 | 7 | 6 | 7 | 6 | 7 | 6 | 7 |
| *P* value | 0.0004 | | 0.001 | | 0.0213 | | 0.0001 | |
| **Liver (mice)** | | | | | | | | |
|  | **Panel K** | | **Panel L** | | **Panel M** | | **Panel N** | |
| Metabolite | **GGT activity** |  | **MDA** | | **Nitrate+Nitrite** | | **Nitrotyrosine/GAPDH** | |
| Data Analysed | **WT** | **Asl^Neo/Neo^** | **WT** | **Asl^Neo/Neo^** | **WT** | **Asl^Neo/Neo^** | **WT** | **Asl^Neo/Neo^** |
| Mean | 3,205 | 15,851 | 0.03 | 0.05 | 0.2 | 0.1 | 0.07 | 0.09 |
| Median | 2,068 | 14,432 | 0.03 | 0.05 | 0.2 | 0.1 | 0.08 | 0.1 |
| SD | 3,158 | 4,957 | 0.009 | 0.02 | 0.1 | 0.03 | 0.03 | 0.03 |
| SEM | 1,579 | 2,479 | 0.003 | 0.009 | 0.04 | 0.01 | 0.01 | 0.01 |
| Lower 95% CI of mean | -1,820 | 7,962 | 0.02 | 0.03 | 0.1 | 0.09 | 0.02 | 0.05 |
| Upper 95% CI of mean | 8,230 | 23,739 | 0.04 | 0.07 | 0.3 | 0.1 | 0.1 | 0.1 |
| n number | 4 | 4 | 6 | 7 | 9 | 7 | 4 | 4 |
| *P* value | 0.0051 | | 0.1091 | | 0.0417 | | 0.3811 | |
| **Liver (mice)** | | | | | | |  |  |
|  | **Panel O** | | **Panel P** | | **Panel Q** | |  |  |
| Metabolite | **GCLC** | | **GCLM** | | **GS** | |  |  |
| Data Analysed | **WT** | **Asl^Neo/Neo^** | **WT** | **Asl^Neo/Neo^** | **WT** | **Asl^Neo/Neo^** |  |  |
| Mean | 3.4 | 1.2 | 4.5 | 1.1 | 1.8 | 1.0 |  |  |
| Median | 3.5 | 1.2 | 4.6 | 1.3 | 1.6 | 1.0 |  |  |
| SD | 0.8 | 0.7 | 1.9 | 0.5 | 0.9 | 0.2 |  |  |
| SEM | 0.4 | 0.3 | 0.9 | 0.2 | 0.4 | 0.1 |  |  |
| Lower 95% CI of mean | 2.0 | 0.1 | 1.4 | 0.3 | 0.3 | 0.6 |  |  |
| Upper 95% CI of mean | 4.7 | 2.2 | 7.7 | 1.9 | 3.3 | 1.4 |  |  |
| N number | 4 | 4 | 4 | 4 | 4 | 4 |  |  |
| *P* value | 0.006 | | 0.01 | | 0.1 | |  |  |

**Table S6B: Descriptive summary of statistical results for Figure 2.**

| **Figure 2** | | | | |
| --- | --- | --- | --- | --- |
|  | **Panel C** | | **Panel D** | |
|  | **FSPG liver retention** | | **FSPG liver retention** | |
| Data Analysed | **WT** | **Asl^Neo/Neo^** | **WT** | **Asl^Neo/Neo^** |
| Mean | 5.2 | 13.6 | 5.3 | 13.4 |
| Median | 4.9 | 12.5 | 5.2 | 14.1 |
| SD | 1.5 | 3.9 | 2.3 | 1.7 |
| SEM | 0.5 | 1.9 | 0.7 | 0.9 |
| Lower 95% CI of mean | 4.1 | 7.4 | 3.7 | 10.6 |
| Upper 95% CI of mean | 6.3 | 19.7 | 7.0 | 16.2 |
| n number | 10 | 4 | 10 | 4 |
| *P* value | <0.0001 | | <0.0001 | |

**Table S6C: Descriptive summary of statistical results for Figure 3.**

| **Figure 3** | | | | | | | | | |
| --- | --- | --- | --- | --- | --- | --- | --- | --- | --- |
|  | **Panel A: Ammonia** | | | | | | | | |
| Timepoint | **2h** | | **24h** | | **72h** | | **7 days** | |  |
| Treatment | Luc mRNA | hASL mRNA | Luc mRNA | hASL mRNA | Luc mRNA | hASL mRNA | Luc mRNA | hASL mRNA | WT |
| Mean | 224 | 511 | 251 | 106 | 461 | 88 | 497 | 101 | 61 |
| Median | 198 | 609 | 282 | 81 | 417 | 87 | 475 | 99 | 57 |
| SD | 175 | 393 | 99 | 76 | 181 | 28 | 192 | 48 | 12 |
| SEM | 101 | 227 | 44 | 29 | 104 | 16 | 96 | 28 | 7 |
| Lower 95% CI of mean | -211 | -465 | 128 | 36 | 11 | 17 | 192 | -18 | 30 |
| Upper 95% CI of mean | 659 | 1,488 | 375 | 177 | 911 | 159 | 803 | 220 | 92 |
| n number | 3 | 3 | 5 | 7 | 3 | 3 | 4 | 3 | 3 |
| *P* value | 0.1 | | 0.5 | | 0.04 | | 0.02 | |  |
|  | **Panel B: ASA** | | | | | | | | |
| Timepoint | **2h** | | **24h** | | **72h** | | **7 days** | |  |
| Treatment | Luc mRNA | hASL mRNA | Luc mRNA | hASL mRNA | Luc mRNA | hASL mRNA | Luc mRNA | hASL mRNA | WT |
| Mean | 118.8 | 1839 | 143.6 | 54.58 | 172.1 | 22.66 | 125.3 | 49.09 | 57 |
| Median | 109.7 | 235.5 | 137.3 | 41.74 | 180.8 | 23.34 | 113.7 | 53.25 | 61 |
| SD | 58.8 | 2824 | 41.64 | 24.3 | 79.35 | 1.181 | 26.08 | 16.22 | 12.49 |
| SEM | 33.95 | 1631 | 18.62 | 10.87 | 45.81 | 0.6817 | 13.04 | 8.11 | 7.211 |
| Lower 95% CI of mean | -27.31 | -5177 | 91.93 | 24.4 | -24.99 | 19.72 | 83.83 | 23.28 | 29.97 |
| Upper 95% CI of mean | 264.9 | 8855 | 195.3 | 84.75 | 369.2 | 25.59 | 166.8 | 74.9 | 92.03 |
| n number | 3 | 3 | 5 | 5 | 3 | 3 | 4 | 4 | 3 |
| *P* value | 0.0178 | | 0.0941 | | 0.0063 | | 0.1834 | |  |
|  | **Panel C: Citrulline** | | | | | | | | |
| Timepoint | **2h** | | **24h** | | **72h** | | **7 days** | |  |
| Treatment | Luc mRNA | hASL mRNA | Luc mRNA | hASL mRNA | Luc mRNA | hASL mRNA | Luc mRNA | hASL mRNA | WT |
| Mean | 254.8 | 407.1 | 300 | 198.1 | 340.6 | 166.7 | 336.8 | 228.1 | 173.8 |
| Median | 239.6 | 418.7 | 278 | 182.8 | 380.4 | 164.3 | 336.3 | 238.9 | 170.7 |
| SD | 73.68 | 53.9 | 63.11 | 25.15 | 122.2 | 5.382 | 11.71 | 42.46 | 6.604 |
| SEM | 42.54 | 31.12 | 28.22 | 11.25 | 70.57 | 3.107 | 5.857 | 21.23 | 3.813 |
| Lower 95% CI of mean | 71.77 | 273.2 | 221.7 | 166.9 | 36.91 | 153.3 | 318.2 | 160.5 | 157.4 |
| Upper 95% CI of mean | 437.8 | 541 | 378.4 | 229.3 | 644.2 | 180 | 355.5 | 295.6 | 190.2 |
| n number | 3 | 3 | 5 | 5 | 3 | 3 | 4 | 4 | 3 |
| *P* value | 0.0134 | | 0.0377 | | 0.0044 | | 0.0503 | |  |
|  | **Panel E** |  |  | **Panel I** | |  |  | | |
| Metabolite | **Orotate** |  |  | **Histology** | |  |  |  |  |
| Treatment | Luc mRNA | hASL mRNA | WT | Luc mRNA | hASL mRNA | WT |  |  |  |
| Mean | 128.2 | 0.994 | 3.433 | 5.024 | 46.15 | 51.11 |  |  |  |
| Median | 95.67 | 0.7074 | 2.33 | 4.719 | 59.19 | 51.35 |  |  |  |
| SD | 121.3 | 0.9655 | 3.272 | 1.041 | 23.23 | 5.447 |  |  |  |
| SEM | 54.27 | 0.4318 | 1.463 | 0.6008 | 13.41 | 3.145 |  |  |  |
| Lower 95% CI of mean | -22.48 | -0.2049 | -0.6298 | 2.439 | -11.55 | 37.58 |  |  |  |
| Upper 95% CI of mean | 278.8 | 2.193 | 7.495 | 7.609 | 103.9 | 64.65 |  |  |  |
| n number | 5 | 5 | 5 | 3 | 3 | 3 |  |  |  |
| *P* value |  | | |  | | |  |  |  |
| WT vs. hASL mRNA | 0.3314 | | | 0.9002 | | |  |  |  |
| WT vs. Luc mRNA | 0.0031 | | | 0.0151 | | |  |  |  |
| hASL mRNA vs. Luc mRNA | 0.0003 | | | 0.0248 | | |  |  |  |
|  | **Panel G: ASL** | | | | | | | | |
| Timepoint | **2h** | | **24h** | | **72h** | | **7 days** | |  |
| Treatment | Luc mRNA | hASL mRNA | Luc mRNA | hASL mRNA | Luc mRNA | hASL mRNA | Luc mRNA | hASL mRNA | WT |
| Mean | 0.08528 | 0.1513 | 0.03525 | 0.9559 | 0.1966 | 0.5688 | 0.2257 | 0.5704 | 1 |
| Median | 0.08866 | 0.1503 | 0.04496 | 0.9521 | 0.1517 | 0.5777 | 0.1724 | 0.4879 | 0.9836 |
| SD | 0.01071 | 0.09662 | 0.01865 | 0.08316 | 0.1424 | 0.2259 | 0.1325 | 0.2158 | 0.3977 |
| SEM | 0.006185 | 0.05578 | 0.01077 | 0.04801 | 0.08223 | 0.1304 | 0.07647 | 0.1246 | 0.2296 |
| Lower 95% CI of mean | 0.05867 | -0.08871 | -0.01107 | 0.7494 | -0.1572 | 0.007577 | -0.1033 | 0.03422 | 0.01201 |
| Upper 95% CI of mean | 0.1119 | 0.3913 | 0.08157 | 1.163 | 0.5504 | 1.13 | 0.5547 | 1.107 | 1.988 |
| n number | 3 | 3 | 3 | 3 | 3 | 3 | 3 | 3 | 3 |
| *p* value | 0.9861 | | <0.0001 | | 0.0112 | | 0.0381 | |  |
|  | **Panel J: ASL activity (fumarate ng/**$\boldsymbol{\mu}$**L)** | | | | | | | | |
| Timepoint | **2h** | | **24h** | | **72h** | | **7 days** | |  |
| Treatment | Luc mRNA | hASL mRNA | Luc mRNA | hASL mRNA | Luc mRNA | hASL mRNA | Luc mRNA | hASL mRNA | WT |
| Mean | 80.69 | 83.2 | 69.82 | 196.6 | 90.52 | 179.3 | 90.17 | 125.7 | 199.2 |
| Median | 83.59 | 81.62 | 69.84 | 195.5 | 87.45 | 203.3 | 93.49 | 121.1 | 198.2 |
| SD | 5.816 | 8.229 | 5.931 | 7.489 | 16.49 | 48.22 | 10.92 | 35.49 | 9.997 |
| SEM | 3.358 | 4.751 | 2.652 | 3.349 | 9.522 | 27.84 | 5.459 | 17.75 | 4.471 |
| Lower 95% CI of mean | 66.24 | 62.76 | 62.46 | 187.3 | 49.55 | 59.5 | 72.8 | 69.2 | 186.8 |
| Upper 95% CI of mean | 95.14 | 103.6 | 77.18 | 205.9 | 131.5 | 299.1 | 107.5 | 182.1 | 211.6 |
| n number | 3 | 3 | 5 | 5 | 3 | 3 | 4 | 4 | 5 |
| *P* value | 0.9998 | | <0.0001 | | 0.0002 | | 0.1043 | |  |

**Table S6D: Descriptive summary of statistical results for Figure 4.**

| **Figure 4** | | | | | | | | | |
| --- | --- | --- | --- | --- | --- | --- | --- | --- | --- |
|  | **Panel B** | | **Panel C** | | |  | | | |
| Parameter | **Survival** | | **Growth** | | |  |  |  |  |
| Treatment | Luc mRNA | hASL mRNA | WT | Luc mRNA | hASL mRNA |  |  |  |  |
| n number | 6 | 5 | 6 | 5 | 4 |  |  |  |  |
|  | | | | | |  |  |  |  |
| *P* value | 0.002 |  | Luc-LNPs vs. ASL-LNPs | | <0.0001 |  |  |  |  |
|  |  |  | Luc-LNPs vs. WT | | <0.0001 |  |  |  |  |
|  |  |  | ASL-LNPs vs. WT | | 0.1222 |  |  |  |  |
|  | **Panel E** | | | **Panel F** | | | **Panel G** | | |
| Metabolite | **Ammonia** | | | **ASA** | | | **Citrulline** | | |
| Treatment | WT | Luc mRNA | hASL mRNA | WT | Luc mRNA | hASL mRNA | WT | Luc mRNA | hASL mRNA |
| Mean | 28.75 | 2954 | 59 | 17.21 | 3123 | 15.73 | 13 | 87.91 | 72.84 |
| Median | 28 | 3125 | 49 | 15.27 | 347.1 | 15.68 | 12.1 | 67.15 | 11.28 |
| SD | 12.15 | 2149 | 27.45 | 4.364 | 6885 | 0.9275 | 2.5 | 72.84 | 2.24 |
| SEM | 6.074 | 877.2 | 12.28 | 2.182 | 2811 | 0.4148 | 1.117 | 27.53 | 1.002 |
| Lower 95% CI of mean | 9.419 | 699.3 | 24.92 | 10.27 | -4102 | 14.58 | 9.9 | 20.54 | 7.552 |
| Upper 95% CI of mean | 48.08 | 5209 | 93.08 | 24.15 | 10348 | 16.88 | 16.1 | 155.3 | 13.09 |
| n number | 4 | 6 | 5 | 4 | 6 | 5 | 4 | 6 | 5 |
| *P* value | | | | | | | | | |
| WT vs. hASL mRNA | 0.9994 | | | >0.9999 | | | 0.9956 | | |
| WT vs. Luc mRNA | 0.0172 | | | 0.5421 | | | 0.0444 | | |
| hASL mRNA vs. Luc mRNA | 0.0124 | | | 0.5008 | | | 0.0371 | | |
|  | **Panel H** | | | **Panel I** | | | **Panel K** | | |
| Metabolite | **Orotate** | | | **C13 urea** | | | **ASL** | | |
| Treatment | WT | Luc mRNA | hASL mRNA | WT | Luc mRNA | hASL mRNA | WT | Luc mRNA | hASL mRNA |
| Mean | 0 | 1207 | 0 | 1.074 | 1.06 | 1.072 | 1 | 0.01717 | 1.364 |
| Median | 0 | 1108 | 0 | 1.074 | 1.06 | 1.071 | 0.7602 | 0.01413 | 1.258 |
| SD | 0 | 622.5 | 0 | 0.004681 | 0.000876 | 0.002905 | 0.4207 | 0.005589 | 0.2646 |
| SEM | 0 | 254.1 | 0 | 0.002341 | 0.000438 | 0.001452 | 0.2429 | 0.003227 | 0.1528 |
| Lower 95% CI of mean | 0 | 554.1 | 0 | 1.066 | 1.059 | 1.067 | -0.04516 | 0.003284 | 0.7071 |
| Upper 95% CI of mean | 0 | 1861 | 0 | 1.081 | 1.076 | 1.061 | 2.045 | 0.03105 | 2.022 |
| n number | 4 | 6 | 5 | 4 | 4 | 4 | 3 | 3 | 3 |
| *P* value | | | | | | | | | |
| WT vs. hASL mRNA | >0.9999 | | | 0.6289 | | | 0.332 | | |
| WT vs. Luc mRNA | 0.0015 | | | 0.0005 | | | 0.0135 | | |
| hASL mRNA vs. Luc mRNA | 0.0009 | | | 0.0015 | | | 0.0029 | | |
|  | **Panel M** | | | **Panel N** | | |  | | |
| Metabolite | **ASL histology** | | | **ASL activity** | | |  |  |  |
| Treatment | WT | Luc mRNA | hASL mRNA | WT | Luc mRNA | hASL mRNA |  |  |  |
| Mean | 85.35 | 12.49 | 91.12 | 309.7 | 77.2 | 310.2 |  |  |  |
| Median | 85.24 | 15.46 | 91.33 | 310.6 | 69.88 | 328.7 |  |  |  |
| SD | 6 | 11.51 | 1.096 | 7 | 17.91 | 29.32 |  |  |  |
| SEM | 2.821 | 5.149 | 0.4902 | 3.843 | 7.312 | 13.11 |  |  |  |
| Lower 95% CI of mean | 76.37 | -1.803 | 89.76 | 293.2 | 58.41 | 273.8 |  |  |  |
| Upper 95% CI of mean | 94.32 | 26.79 | 92.48 | 326.2 | 96 | 346.6 |  |  |  |
| n number | 4 | 5 | 5 | 4 | 6 | 5 |  |  |  |
| *P* value | | | | | | |  |  |  |
| WT vs. hASL mRNA | 0.513 | | | 0.513 | | |  |  |  |
| WT vs. Luc mRNA | <0.0001 | | | <0.0001 | | |  |  |  |
| hASL mRNA vs. Luc mRNA | <0.0001 | | | <0.0001 | | |  |  |  |

**Table S6E: Descriptive summary of statistical results for Figure 5.**

| **Figure 5** | | | | | | | | | |
| --- | --- | --- | --- | --- | --- | --- | --- | --- | --- |
|  | **Panel B** | | **Panel C** | | |  | | | |
| Parameter | **Survival** | | **Growth** | | |  |  |  |  |
| Treatment | Luc mRNA | hASL mRNA | WT | Luc mRNA | hASL mRNA |  |  |  |  |
| n number | 6 | 7 | 6 | 7 | 6 |  |  |  |  |
|  | | | | | |  |  |  |  |
| p value | 0.002 |  | Luc-LNPs vs. ASL-LNPs | | <0.0001 |  |  |  |  |
|  |  |  | Luc-LNPs vs. WT | | <0.0001 |  |  |  |  |
|  |  |  | ASL-LNPs vs. WT | | 0.00913 |  |  |  |  |
|  | **Panel E** | | | **Panel F** | | | **Panel G** | | |
| Metabolite | **Ammonia** | | | **ASA** | | | **Citrulline** | | |
| Treatment | WT | Luc mRNA | hASL mRNA | WT | Luc mRNA | hASL mRNA | WT | Luc mRNA | hASL mRNA |
| Mean | 71 | 338 | 74 | 0 | 192.7 | 28.9 | 16.6 | 224 | 31.3 |
| Median | 48 | 345 | 55 | 0 | 259.7 | 12.8 | 16.9 | 233 | 21.1 |
| SD | 5 | 205 | 52 | 0 | 192.7 | 36.2 | 2.5 | 44.8 | 10.3 |
| SEM | 19 | 92 | 21 | 0 | 86.2 | 14.8 | 0.9 | 20.0 | 4.2 |
| Lower 95% CI of mean | 26 | 82 | 19 | 0 | 86.3 | -8.9 | 14.5 | 168.4 | 20.4 |
| Upper 95% CI of mean | 116 | 593 | 129 | 0 | 564.8 | 66.9 | 18.8 | 279.7 | 42.1 |
| N number | 8 | 5 | 6 | 10 | 5 | 6 | 8 | 6 | 5 |
| *P* value | | | | | | | | | |
| WT vs. hASL mRNA | 01.0 | | | 0.8 | | | 0.5 | | |
| WT vs. Luc mRNA | 0.002 | | | <0.0001 | | | <0.0001 | | |
| hASL mRNA vs. Luc mRNA | 0.004 | | | 0.0001 | | | <0.0001 | | |
|  | **Panel H** | | | **Panel I** | | | **Panel K** | | |
| Metabolite | **Orotate** | | | **C13 urea** | | | **ASL levels** | | |
| Treatment | WT | Luc mRNA | hASL mRNA | WT | Luc mRNA | hASL mRNA | WT | Luc mRNA | hASL mRNA |
| Mean | 0.7 | 18.8 | 3.2 | 1.089 | 1.077 | 1.086 | 1 | 0.1002 | 1.045 |
| Median | 0.07 | 9.9 | 0.7 | 1.089 | 1.078 | 1.086 | 0.9699 | 0.8773 | 1.044 |
| SD | 1.2 | 18.1 | 6.7 | 0.004218 | 0.000957 | 0.004355 | 0.1765 | 0.04394 | 0.5783 |
| SEM | 0.4 | 8.1 | 2.7 | 0.001334 | 0.000479 | 0.001778 | 0.1019 | 0.01794 | 0.2361 |
| Lower 95% CI of mean | -0.3 | -3.6 | -3.8 | 1.086 | 1.076 | 1.081 | 0.5614 | 0.0541 | 0.4383 |
| Upper 95% CI of mean | 1.7 | 41.2 | 10.2 | 1.092 | 1.079 | 1.09 | 1.439 | 0.1463 | 1.652 |
| n number | 8 | 5 | 6 | 10 | 4 | 6 | 3 | 6 | 6 |
| *P* value | | | | | | | | | |
| WT vs. hASL mRNA | 0.9 | | | 0.3 | | | 0.9 | | |
| WT vs. Luc mRNA | 0.01 | | | 0.0003 | | | 0.01 | | |
| hASL mRNA vs. Luc mRNA | 0.04 | | | 0.009 | | | 0.003 | | |
|  | **Panel M** | | |  | | | | | |
| Metabolite | **ASL activity** | | |  |  |  |  |  |  |
| Treatment |  |  |  |  |  |  |  |  |  |
| Mean | 246.5 | 89.1 | 181.7 |  |  |  |  |  |  |
| Median | 247.3 | 81.9 | 179.2 |  |  |  |  |  |  |
| SD | 16 | 15.8 | 67.9 |  |  |  |  |  |  |
| SEM | 5.229 | 7.06 | 27.7 |  |  |  |  |  |  |
| Lower 95% CI of mean | 234.5 | 69.5 | 110.4 |  |  |  |  |  |  |
| Upper 95% CI of mean | 258.6 | 108.8 | 253 |  |  |  |  |  |  |
| n number | 9 | 5 | 6 |  |  |  |  |  |  |
| *P* value | | | |  |  |  |  |  |  |
| WT vs. hASL mRNA | 0.01 | | |  |  |  |  |  |  |
| WT vs. Luc mRNA | <0.0001 | | |  |  |  |  |  |  |
| hASL mRNA vs. Luc mRNA | 0.003 | | |  |  |  |  |  |  |

**Table S6F: Descriptive summary of statistical results for Figure 6.**

| **Figure 6** | | | | | | | | | |
| --- | --- | --- | --- | --- | --- | --- | --- | --- | --- |
|  | **Panel B** | | | **Panel C** | | | | **Panel D** | |
| Metabolite | **FSPG liver retention** | | | **Total glutathione in liver** | | | | **Total Homocysteine in liver** | |
| Treatment | WT | Untreated | hASL mRNA | WT | Luc mRNA | hASL mRNA neonatal | hASL mRNA adult | Untreated | hASL mRNA |
| Mean | 5.0 | 21.7 | 11.2 | 0.1 | 0.03 | 0.08 | 0.09 | 2.7 | 1.2 |
| Median | 4.7 | 22.4 | 11.0 | 0.1 | 0.03 | 0.08 | 0.09 | 2.4 | 1.2 |
| SD | 2.7 | 2.3 | 1.9 | 0.009 | 0.02 | 0.02 | 0.006 | 0.8 | 0.2 |
| SEM | 1.4 | 1.1 | 0.9 | 0.005 | 0.009 | 0.01 | 0.003 | 0.3 | 0.1 |
| Lower 95% CI of mean | 0.6 | 18.0 | 8.1 | 0.1 | -0.003 | 0.05 | 0.08 | 1.9 | 0.9 |
| Upper 95% CI of mean | 9.4 | 25.4 | 14.2 | 0.1 | 0.06 | 0.1 | 0.1 | 3.4 | 1.5 |
| n number | 4 | 4 | 4 | 4 | 4 | 4 | 4 | 7 | 5 |
| *P* value | | | | | | | | | |
| WT vs. Untreated | <0.0001 | | | WT vs. Luc mRNA | | | <0.0001 | n/a | |
| WT vs. hASL mRNA | 0.005 | | | WT vs. hASL mRNA (Neonatal) | | | 0.06 | n/a | |
| Untreated vs. hASL mRNA | 0.0003 | | | WT vs. hASL mRNA (Adult) | | | 0.2 | 0.002 | |
|  |  |  |  | Luc mRNA vs. hASL mRNA (Neonatal) | | | 0.0007 |  |  |
|  |  |  |  | Luc mRNA vs. hASL mRNA (Adult) | | | 0.0002 |  |  |
|  |  |  |  | hASL mRNA (Neonatal) vs. hASL mRNA (Adult) | | | 0.8 |  |  |

**Table S6G: Descriptive summary of statistical results for Figure 7.**

| **Figure 7** | | | | |
| --- | --- | --- | --- | --- |
|  | **Panel F** | | **Panel G** | |
|  | **GCLC Fold difference** | | **GCLM Fold difference** | |
| Treatment | **DMSO** | **SNAP** | **DMSO** | **SNAP** |
| Mean | 1 | 1.7 | 1 | 1.6 |
| Median | 1 | 1.7 | 1 | 1.6 |
| SD | 0 | 0.3 | 0 | 0 |
| SEM | 0 | 0.1 | 0 | 0.03 |
| Lower 95% CI of mean | 1 | 1.0 | 1 | 1.3 |
| Upper 95% CI of mean | 1 | 2.3 | 1 | 1.8 |
| N number | 3 | 3 | 3 | 3 |
| *P* value | 0.0004 | | 0.01 | |

**Table S6: Summarised statistical results for main figures 1-7.** Tables include mean, median, SD, SEM, Lower and upper 95% CI, n numbers and *p*-values post statistical test comparison per groups. SD= standard deviation, SEM= standard error of mean, CI= confidence interval.

**Table S7: List of primary and secondary antibodies**

| **Antibodies** | **Host** | **Company** | **Catalog num** | **Dilution used** |
| --- | --- | --- | --- | --- |
| Anti-ACSL4 [F-4] | Rabbit | Santa Cruz Biotechnology | Sc-365230 | 1:200 WB |
| Anti-Actin | Rabbit | Cell Signalling | 4967 | 1:1000 WB |
| Anti-ASL | Rabbit | Abcam | Ab97370 | 1:1000 WB and histology |
| Anti-GAPDH | Mouse | Abcam | Ab97370 | 1:10000 WB |
| Anti-GPX4 | Rabbit | Abcam | Ab125066 | 1:500 WB |
| Anti-Nrf2 (fibroblast) | Rabbit | ThermoFisher | **PA5-27882** | 1:500 WB |
| Anti-Nrf2 (liver) | Rabbit | Abcam | Ab62352 | 1:500 WB |
| Anti-GAPDH | Mouse | Abcam | Ab97370 | 1:10000 WB |
| Anti-xCT | Rabbit | Novus Biologicals | NB300-318 | 1:500 WB |
| Anti-Nitrotyrosine | Mouse | Merck Millipore | 05-233 clone 1A6 | 1:100 WB |
| Anti-GAPDH | Rabbit | Abcam | Ab9485 | 1:1000 WB |
| IRDye 800CW goat anti-rabbit IgG |  | Licor | 926-32210 | 1:1000 in-cell  1:10,000 WB |
| IRDye 680RD Donkey anti-mouse IgG |  | Licor | 923-68072 | 1:10,000 WB |
| CellTag 700 |  | Licor | 926-41091 | 1:1000 |
| HRP-linked anti-rabbit IgG |  | Cell Signalling | 7074 | 1:200 WB |

**Table S8: List of materials and reagents**

| **Name** | **Company** | **Catalog number** |
| --- | --- | --- |
| 1,1,3,3-Tetramethoxypropane (MDBMA) | Sigma-Aldrich | 108383-100ML |
| 2,3,4,5,6-Pentafluorobenzyl bromide (PFBBr) | Sigma-Aldrich | 101052-5G |
| 2-β-mercaptoethanol (β-ME), | Bio-rad | 1610710 |
| 4x Laemmli sample buffer | Bio-Rad | 1610747 |
| Ammonium Formiate | Sigma-Aldrich | 156264 |
| Argininosuccinic Acid disodium salt hydrate | Sigma | A5707 |
| BCA Kit | ThermoFisher | 23227 |
| CellBind 96 well microplates | VWR | 66025-626 |
| Chameleon Duo Pre-stained Protein Ladder | Licor | 928-60000 |
| D-luciferin firefly | Gold Biotechnology | L-123-10 |
| Dichloromethane | VWR Chemicals | 23366.293 |
| DMEM | ThermoFisher | 41965-039 |
| [DPBS, no calcium, no magnesium-10 x 500 mL](https://prilon5web2.sci-ware-customer.com/sword/catalogueItem.do?key=1356344&context=PO16225690) | ThermoFisher | 14190169 |
| EDTA-free proteinase inhibitor cocktail | Roche | 11836170001 |
| ECL Prime Western Blotting Detection Reagent | Cytiva | RPN2236 |
| FBS (Heat-Inactivated) | Sigma | F9665 |
| Fumarate kit | Abcam | Ab102516 |
| Formic Acid 99% Optima LC/MS grade | Fisher chemical | A117-50 |
| FUJI DRI-CHEM SLIDE NH3-PIIS | Fujifilm | 15809633 |
| FUJI DRI-CHEM SLIDE ALT/GPT-PIIS | Fujifilm | 16654035 |
| Gamma glutamyl transferase (GGT) Assay Kit (Colorimetric) | Abcam | Ab241029 |
| Hexane | Sigma-Aldrich | 34859-2.5L |
| HPLC grade Acetonitrile | Fisher Chemical | 10660131 |
| HPLC grade Methanol | Fisher chemical | 10675112 |
| Hydrochloric acid | BDH | 101254H |
| Licor Blocking buffer | Licor | 927-40000 |
| Luminescent-based GSH/GSSG-Glo Assay Kit | Promega | V6611 |
| Lysing Matrix D tube | MP Biomedicals |  |
| Phosphate Buffer Solution 1M pH 7.4 | Sigma-Aldrich | P3619-1GA |
| Polink-2 Plus HRP Polymer and AP Polymer detection for Rb antibody kit | Origene | D39-18 |
| Potassium dihydrogen phosphate | VWR Chemicals | 153184U |
| Precellys ceramic-kit 1.4/2.8 mm 2ml | VWR International | 431-0710 |
| PVDF membrane 0.45um | GE healthcare | 15259894 |
| Tetrabutylammonium bisulphate (TBA) | Sigma-Aldrich | 86868-25G |
| Qiagen RNeasy kits | Qiagen | 74004 |
| L-Arginine | Sigma-Aldrich | A5006 |
| Argininosuccinic Acid (ASA) | Sigma-Aldrich | A5707 |
| L-Citrulline | Sigma-Aldrich | C7629 |
| L-Glutamic Acid | Sigma-Aldrich | G1251 |
| L-Glutamine | Sigma-Aldrich | G3126 |
| Ornithine | Sigma-Aldrich | O2375 |
| Creatinine | Sigma | C4255 |
| Orotic Acid | Sigma | O2750 |
| Urea-^13^C | Sigma-Aldrich | 299359-1G |
| L-Arginine-13C6 | CK Isotopes | CLM-2265-H |
| L-Citrulline-d7 | CDN Isotopes | D-7306 |
| L-Glutamic Acid-d5 | CDN Isotopes | D-0899 |
| L-Glutamine-13C2 | CK Isotopes | CLM-2001 |
| L-Ornithine-d7 HCl | CDN Isotopes | D-7319 |
| 1,3-15N2 orotic acid | Cambridge Isotope laboratories | NLM-1048-PK |
| Creatinine (N-Methyl-D3, 98%) | CDN Isotopes | D-3689 |
| Urea-^13^C | Sigma-Aldrich | 299359-1G |
| Creatinine (N-Methyl-D3, 98%) | CDN Isotopes | D-3689 |
| Urea-^13^C | Sigma-Aldrich | 299359-1G |
| Acquity UPLC BEH Amide column (2.1x100mm, 1.7µm particle size) | Waters | 186004801 |
| Van Guard UPLC BEH Amide pre-column (2.1x5mm, 1.7µm particle size) | Waters | 186004799 |
| ACQUITY UPLC BEH C18 Column, 130Å, 1.7 µm, 2.1 mm X 50 mm, 1/pk | Waters | 186002350 |

**Table S9: List of instruments**

| **Instrument name** | **Company** |
| --- | --- |
| Capintech dose calibrator | Mirion medical |
| Concentrator plus | Eppendorf |
| DRI-CHEM NX600 | Fujifilm |
| FLUOstar Optima | BMG Labtech |
| GCMS Instrument | Thermoscientific |
| iBright Imaging System | ThermoFisher Scientific |
| IVIS Spectrum In Vivo Imaging System | PerkinElmer |
| In cell western Fluorescence Imaging scanner | Licor Odyssey CLx |
| NanoPET/CT plus system | Mediso |
| Precellys 24 tissue homogeniser | Bertin instruments |
| Precellys Evolution | Bertin Technologies |
| Xevo TQ-S | Waters |
| Zeiss Axioplan | Zeiss |

The following Excel files are available online:

**Data file S1. Raw, individual-level data for experiments for main text figures 1 to 7 and figures S1 to S11.**

**Data file S2. Liver untargeted proteomic dataset from Asl^Neo/Neo^ mice and WT littermates.**
